# Seagrass and oyster interactions under a warming climate scenario: A mesocosm experiment

**DOI:** 10.1371/journal.pone.0337843

**Published:** 2025-12-11

**Authors:** Natalie Schafer, Suzanne Ayvazian, Cathleen Wigand, Kenneth Miller, Donald Cobb, Susanna Osinski

**Affiliations:** 1 ORAU Student Services Contractor, U.S. Environmental Protection Agency, Office of Research and Development, Center for Environmental Measurement and Modeling, Atlantic Coastal Environmental Science Division, Narragansett, Rhode Island, United States of America; 2 U.S. Environmental Protection Agency, Office of Research and Development, Center for Environmental Measurement and Modeling, Atlantic Coastal Environmental Science Division, Narragansett, Rhode Island, United States of America; 3 General Dynamics Information Technology, Falls Church, Virginia, United States of America; 4 ORISE Fellow, U.S Environmental Protection Agency, Office of Research and Development, Center for Environmental Measurement and Modeling, Atlantic Coastal Environmental Science Division, Narragansett, Rhode Island, United States of America; The University of Sydney School of Biological Sciences: The University of Sydney School of Life and Environmental Sciences, AUSTRALIA

## Abstract

Seagrass and oyster beds are biogenic habitats of global importance in shallow coastal waters as they provide critical ecosystem functions. These habitats may be adversely affected by warming trends. Short-term effects and interactions of oysters (presence, absence) and water temperature (ambient or warmed +2 °C) on eelgrass, macroalgae, chlorophyll *a* concentration, and water quality were simulated in mesocosms. Eelgrass (*Zostera marina*) biomass and chlorophyll *a* concentrations were significantly reduced under elevated water temperature. There were no short-term (9-weeks) effects of oyster (*Crassostrea virginica*) presence on eelgrass biomass, chlorophyll *a* concentration, nutrients, dissolved inorganic carbon, or dissolved organic carbon. Hourly records of water temperature and light showed an apparent oyster effect with significantly lowered temperatures in the warmed mesocosms and increased light transmittance in warmed and ambient mesocosms for some parts of the day. Surface-only macroalgae were significantly greater in the oyster mesocosms under both warmed and ambient conditions at the end of the experiment than in the eelgrass only mesocosms. In addition, macroalgal coverage (3-dimensional estimate) throughout the mesocosm showed a greater presence in the mesocosms with both oysters and elevated temperatures. A possible outcome of this finding was attenuation of warming, possibly by shading, in the warmed mesocosms. By extension, interactions of oysters and macroalgae may play a role in mitigating warming water temperature in some shallow coastal habitats, highlighting the importance of considering the interactive effects of seagrass and oysters, and other biotic components such as macroalgae, when investigating climate impacts on coastal ecosystems.

## Introduction

Seagrass beds and oyster reefs are two shallow coastal biogenic habitats of global importance and provide many critical ecosystem functions, including habitat provisioning for the maintenance of diversity of the early life history stages of many commercially and recreationally important finfish and shellfish species [1], reduction of turbidity of coastal waters through particle removal, and reduction of wave energy to adjacent shorelines [2]. Yet these critical shallow water coastal habitats have experienced dramatic global depletion due to multiple anthropogenic factors including pollution, urbanization, eutrophication, overfishing, and warming waters due to climate change. As a result, there has been a global loss of 29% of seagrass habitat and 85% of oyster reefs in approximately 150 years [3–6]. Despite their minor global footprint of 0.2%, seagrass beds and underlying sediments store about 10% of the carbon buried in the oceans each year [7,8].

The addition of excess nutrients to aquatic systems fuels increased production of fast-growing algae and epiphytes on leaves attenuating ambient light in the water column, leading to the loss of seagrasses [9,10]. Previous research indicates that co-locating oysters, or other suspension-feeding bivalves, with seagrasses, will improve water clarity by conveying phytoplankton and nutrients from the water column through the bivalve filter feeding process, transferring feces and pseudofeces (biodeposits) to the benthos and increasing sediment porewater nutrients to fertilize the seagrass [11]. This process removes particulates from the water column improving light levels and increases the availability of nutrients in sediments to enhance seagrass productivity [12,13].

Historically, the eastern oyster, *Crassostrea virginica,* provided a productive and profitable fishery along the eastern Atlantic coastline [14]. Oyster populations are credited with improving nutrient reductions through their filter feeding and denitrification activities [15,16], increasing suitable benthic substrate for invertebrates and fishes [17,18], enhancing the productivity of seagrass meadows through improving light availability and providing nutrient rich biodeposits [19,20], and attenuating wave activity and providing shoreline protection [21–23]. The restoration of ecosystem services provided by both oyster and seagrass habitats is the rationale for management efforts to protect and restore these foundation species.

In addition to the factors which have limited seagrass distribution in the past, one of the greatest current and future threats to the sustainability of seagrass populations is increasing sea surface temperatures (SST) in northeastern Atlantic estuaries [24]. The Northeast shelf SST is projected to rise 0.42 °C per decade through the 21^st^ century [25]. This increase in temperature can be primarily attributed to anthropogenic activities that drive climate change [26]. Increasing water temperatures have already been recorded in multiple estuaries along the northeastern Atlantic coastline. Water temperatures have increased by 1.4–1.6 °C in Narragansett Bay, Rhode Island since the 1960’s [27] with a similar temperature increase observed in Woods Hole, Massachusetts [28], the Hudson River Estuary, New York [29], and the Chesapeake Bay estuary, Maryland and Virginia [30,31]. Warmer water temperatures during summer months may be magnified in shallow water estuaries thereby threatening growth and extent and distribution of temperate eelgrass, *Zostera marina* on both the Northeast and Northwest US coastlines [24,32,33]. Increasing SST is also impacting the phenology and biomass of phytoplankton the primary food for oysters with phytoplankton biomass declining by 50% between 1968 and 2019 in Narragansett Bay, Rhode Island [34].

Where present in combination, the occurrence of oysters can affect seagrass ecosystems, yet it is unknown how this may be altered in the future with warming waters. To address this knowledge gap, a short term mesocosm experiment was designed to simulate a temperate coastal salt pond system vegetated with eelgrass to examine the interactive effects between oysters (presence, absence) and temperature (ambient, warmed) on eelgrass growth, macroalgal cover, chlorophyll, light, and water quality. Multiple mesocosm experiments have been designed to examine climate change impacts and suggest that while there are caveats concerning the direct extrapolation of results from mesocosm studies to a field setting, designing a controlled study is exceedingly beneficial to quantify responses between study organisms [35].

The aims of the research were to address the following hypotheses. Warming water temperatures would cause declines in oyster and seagrass biomass, but increase macroalgal cover, reducing light availability to seagrass. Additionally, the presence of oysters in mesocosms would reduce chlorophyll *a* concentrations, increase light, and improve water quality allowing enhanced growth of eelgrass. Lastly, the interaction of oysters and warming temperatures will amplify declines of eelgrass and increases of macroalgal cover. The results from this research may inform both future restoration of eelgrass in coastal areas with oyster reefs or aquaculture and the proximity of shellfish aquaculture leases to current or historic eelgrass beds.

## Methods

### Experimental design

A short-term (9-week), controlled mesocosm study using a 2 x 2 factorial design was used to quantify main effects and interactions between oyster (presence, absence) and temperature (ambient, warmed + 2 °C) treatments (n = four replicates) on eelgrass (*Zostera marina*) growth and persistence, macroalgae growth, water quality factors, chlorophyll *a* and nutrient concentrations. Each of the 16 mesocosms is considered an experimental unit and treatment type was randomly assigned to each mesocosm. Each mesocosm contained eelgrass shoots. Four replicate mesocosms were included for each of the four treatments (eelgrass + oysters present at ambient temperature, eelgrass + oysters present at warmed temperature, eelgrass only at ambient temperature, and eelgrass only at warmed temperature).

The 435 L fiberglass mesocosms are (82 cm wide x 70 cm deep) housed in the greenhouse at the U. S. EPA Atlantic Coastal Environmental Sciences Division (ACESD) in Narragansett, RI. The mesocosms each received raw seawater from Narragansett Bay, R. I. Ambient temperature seawater (averages 20.5 °C June and 24.2 °C August) is pumped from the bay into header troughs. The troughs have overflow drains and there is a constant flow in the troughs of incoming and overflowing water. The seawater to be heated is diverted and accumulates in a separate trough which is heated by a heat exchanger set to the experimental temperature. Once again, this warmed water is always flowing. Ambient temperature seawater supplied eight mesocosms while water warmed by approximately 2 °C greater than ambient temperatures supplied eight treatment mesocosms (Table 1). The primary reason for choosing plus 2 °C was to simulate present-day warming observed in Narragansett Bay and the expected temperature increases in future decades. Mesocosms experienced a diurnal tide cycle each day including two high tides (9:00 am and 9:00 pm) and two low tides (3:00 am and 3:00 pm). The high tide in the mesocosms was approximately 70 cm and low tide approximately 60 cm, resulting in a tidal range of approximately 10 cm. This tidal range allowed for the maximum water depth for the growth of eelgrass shoots. The water took approximately two hours to fill for high tide or drain for low tide. The water levels were maintained for four hours before the next tide cycle was initiated. This schedule did not mimic the natural diurnal cycle which changes daily. The timing of this cycle was set with an automated system. Salinity values of the incoming seawater ranged from 31.2 ppt during high tides in June to 33.0 ppt during low tides in July, and pH values ranging from 7.6 at ambient temperatures during low tides in July to 8.5 at ambient temperatures during low tides in August. In mid-July, after approximately five weeks, all mesocosms had excess macro- and filamentous algae removed from the surface of the tank and around the water inflow and outflow drains to prevent clogging of the water system.

**Table 1:** Nutrient concentrations and environmental variables (mean ± se) at high and low tidal stage and ambient (n = 8) and warmed (n = 8) water temperatures in June and August. There were no statistically significant effects of the presence of oysters on the values of the environmental variables, therefore results have been combined.

	Month	High Tide		Low Tide	
Variable	2021	Ambient	Warmed	Ambient	Warmed
Temperature	June	20.66 ± 0.14	22.06 ± 0.09	19.51 ± 0.08	20.74 ± 0.07
Temperature	August	24.15 ± 0.12	25.91 ± 0.15	22.44 ± 0.53	24.08 ± 0.52
DIC µM	June	24.09 ± 0.05	23.75 ± 0.09	24.12 ± 0.03	23.88 ± 0.12
DIC µM	August	21.52 ± 0.76	23.95 ± 0.31	21.06 ± 0.92	23.99 ± 0.08
DOC µM	June	2.70 ± 0.15	3.63 ± 0.23	4.35 ± 0.93	3.05 ± 0.13
DOC µM	August	5.26 ± 0.35	5.80 ± 0.28	3.54 ± 0.46	4.13 ± 0.38
NH_3_ µM	June	4.89 ± 0.25	5.78 ± 0.47	6.57 ± 0.45	4.86 ± 0.33
NH_3_ µM	August	6.13 ± 0.40	6.95 ± 1.40	5.22 ± 0.43	6.13 ± 0.47
NO_2-_ µM	June	0.77 ± 0.07	0.74 ± 0.07	0.92 ± 0.03	0.65 ± 0.03
NO_2-_ µM	August	0.59 ± 0.03	0.65 ± 0.06	0.83 ± 0.08	0.88 ± 0.08
NO_3-_ µM	June	0.41 ± 0.10	0.60 ± 0.15	0.36 ± 0.07	0.61 ± 0.15
NO_3-_ µM	August	1.06 ± 0.24	1.71 ± 0.44	1.45 ± 0.29	1.10 ± 0.26
PO_4_^3-^ µM	June	1.11 ± 0.10	1.07 ± 0.11	1.45 ± 0.05	0.99 ± 0.06
PO_4_^3-^ µM	August	0.70 ± 0.06	0.84 ± 0.11	1.00 ± 0.08	1.11 ± 0.04
DO (%)	June	100.69 ± 1.13	107.29 ± 2.41	97.93 ± 0.56	104.83 ± 1.29
DO (%)	July			89.23 ± 7.57	102.06 ± 2.05
DO (%)	August			128.90 ± 0.30	105.37 ± 5.09
Chl *a* (µg/L)	June	1.95 ± 0.10	1.31 ± 0.14	1.85 ± 0.06	1.42 ± 0.33
Chl *a* (µg/L)	August	5.31 ± 0.90	4.37 ± 0.82	2.18 ± 0.47	2.15 ± 0.47

Hobo pendant temperature and light loggers (Onset, Bourne, MA) were attached to overflow standpipes at ~39 cm from the bottom of the mesocosm with the sensor positioned upwards to collect temperature and light data each day from June 9, 2021 to August 4, 2021 between the hours of 5:30 am and 9:00 pm at 30-minute intervals. A YSI Professional Plus water sampler (YSI, Yellow Springs, Ohio) was used to measure June (two weeks post-initiation) and August (nine weeks post-initiation) dissolved oxygen and temperature at both high and low tidal cycles.

**Fig 1:**
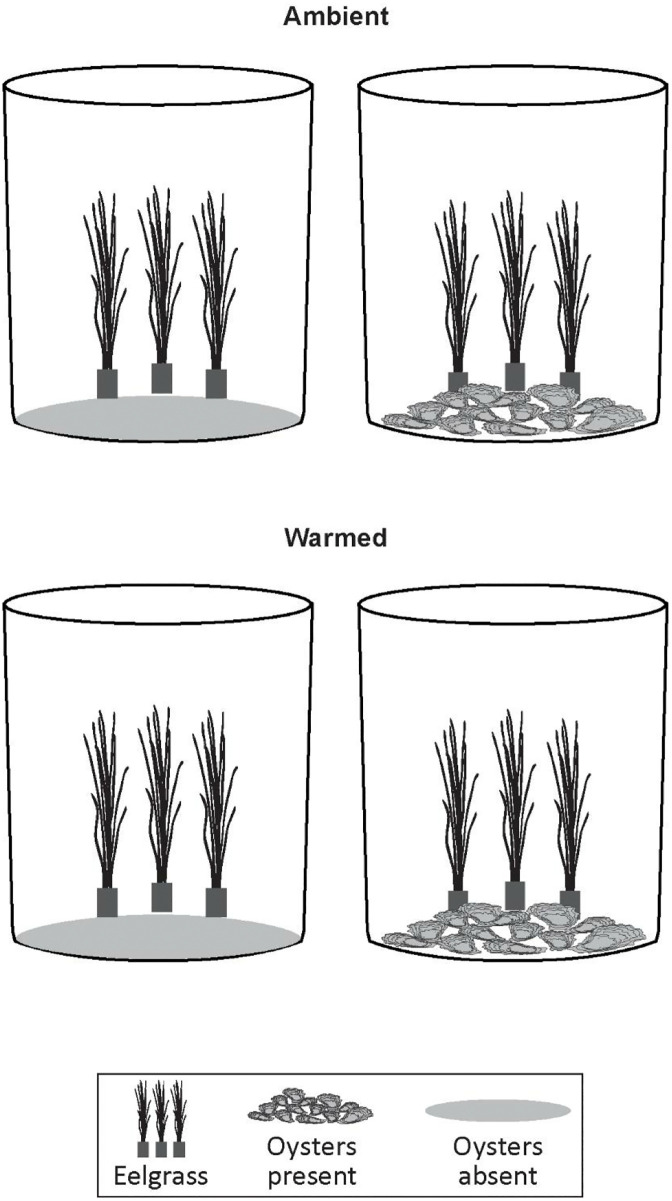
Experimental design. The 2 x 2 experimental design included temperature (ambient and warmed water) and oyster (presence, absence) treatments (n = 4 each), and eelgrass, *Zostera marina* included in each of the 16 mesocosms. In each mesocosms, eelgrass shoots were contained in pots and in eight mesocosms 28-31 oysters were placed on the bottom of the mesocosm adjacent to the eelgrass plant pots.

The non-reproductive eelgrass shoots and sediment were collected by hand with a shovel from Potter Pond, RI (41.3948° N, 71.5375° W) and distributed into 48 plastic plant pots (5 cm width x 20 cm) in June 2021. Fine sand (approximately 0.5 cm) collected from the site was added to the surface of each plant pot to stabilize the sediments. Three pots were randomly selected and centered on one side of each of 16 mesocosms (Fig 1).

In each mesocosm the total number of living eelgrass shoots were recorded and shoot lengths measured after two weeks in mid-June (two weeks post initiation) and in early August (nine weeks after initiation). Shoots were measured from the top of the sheath to the tip of the longest leaf. At the end of the experiment, aboveground living shoots and belowground roots and rhizomes from each mesocosm were harvested and dried at 60 °C to determine dry weight biomass (g). Two visual estimation approaches were used to estimate total floating macro- and filamentous algae (including colonial diatoms) extent at high tide in each mesocosm in August. Species of macro- and filamentous algae were not differentiated. Two researchers estimated the percent cover of macro- and filamentous algae in each mesocosm as one of the five Braun–Blanquet cover-class categories in each assessment approach and the average of the two researchers was reported [36]. In the first assessment approach, surface percent cover of algae was estimated, and in the second assessment approach cover of macro-and filamentous algae was based on estimating the 3 – dimensional (3 – D) percent cover in each mesocosm. As a proxy for algal colonization, we estimated percent algal cover on the wall of the mesocosm between the low and high tide line.

Oysters (*C. virginica*) were collected by hand from a remnant native population in Green Hill Pond, RI (41.3710° N, 71.6132° W) in May 2021. In the lab, oyster shells were scrubbed to remove excess epiphyte cover, measured for total shell height (mm), and allowed two weeks to acclimate in ambient sea water before 28–31 oysters were added to each of eight oyster treatment mesocosms. The variability in the number of oysters per mesocosm results from occasions when small oysters were attached to a larger oyster shell. The initial average oyster height was 61.67 mm (se ± 1.27) and average live wet weight was 66.25 g (se ± 19.21). In the eight oyster treatment mesocosms, the oysters were co-located on the bottom of the mesocosm opposite the eelgrass plant pots (Fig 1). At the end of the experiment, oysters were weighed for total wet weight (g). A sub-sample of five oysters from each of the eight mesocosms (n = 40) were shucked and soft tissue and shell were dried at 60 °C and reweighed to determine dry biomass (g). At weeks five and nine post-initiation, each oyster was removed from their respective mesocosm and the epiphytes scraped off their shells with a clean metal spatula into a labelled pre-weighed aluminum pan. Aluminum pans were placed in a drying oven for 24 hours at 60 °C, removed, and reweighed to obtain a dry biomass of epiphyte (mg).

Chlorophyll *a*, dissolved nutrients (ammonia (NH_3_), nitrite (NO_2-_), nitrate + nitrite (NO_x_), and ortho-phosphate (PO_4_^3-^)) and dissolved inorganic carbon (DIC) and dissolved organic carbon (DOC) concentrations were measured in mid-June (two weeks post initiation) and early August (9 weeks post initiation) in each mesocosm at both high and low tidal conditions. Chlorophyll *a* concentrations were assessed by collecting 60 mL of seawater from each mesocosm in an acid-stripped 60 mL syringe and passing it through a Swinnex filter tip containing a glass fiber filter (Whatman GF/F 0.7 micron). The filter paper was removed from the Swinnex filter tip, wrapped in aluminum foil, placed in a labelled plastic bag on ice and stored in the freezer prior to an acetone extraction and analyzed within two weeks of collection on the Turner Trilogy Fluorometer (San Jose, CA, USA). Water samples for dissolved nutrients were collected from each mesocosm with a 60 mL syringe and filtered through a GF/F filter, stored in acid-washed plastic 20 mL scintillation vials, and immediately frozen until analysis on an Astoria-Pacific Astoria 2 continuous-flow analyzer (Astoria-Pacific, Clackamas, OR). Standard analytical methods (US EPA methods 353.2 and 350.1) were used to determine concentrations of each nutrient, and a five-point standard curve, with check standards run every 15 samples and Milli-Q blanks every 10 samples were utilized. Sample water was filtered through a GF/F filter for measuring DOC and collected raw for DIC, and preserved with 20 µL of mercuric chloride, before determining DIC and DOC concentrations on a Shimadzu Total Organic Carbon Analyzer (Shimadzu Co., Columbia, MD).

### Statistical analyses

Statistical analyses were done separately for high and low tide periods for the YSI measurements, nutrient, chlorophyll, and Hobo data. This was done due to differences in sampling frequencies between the two periods, such as only having July data during low tidal periods. This imbalance impacted direct comparisons between measurements collected during the two periods when combining data across tidal periods. As plant and algae metrics were only measured at high tide, tidal period was not considered in the statistical analyses. For measurements for which both oysters and temperature were varied experimentally, multi-factor ANOVA models were used to assess the main effects of temperature, oysters, and an oyster by temperature interaction on eelgrass and macroalgae. For the multi-factor ANOVA models there were n = 4 replicates for each treatment unless otherwise stated. When the sample collection included multiple months, the sampling month was included as an additional factor, and interactions between month and temperature and oysters were also included. When the interaction was significant, pairwise comparisons using a Bonferroni correction were used to test for differences. For the individual eelgrass shoot length comparisons, the experimental mesocosm was included as an additional nested factor. For all measurements, other than DIC and temperature, results were natural log-transformed prior to fitting the statistical models to better meet the assumptions of parametric statistical tests. We report means ± standard error (± se). Probability for significance for all analyses was set at p < 0.05, unless otherwise stated.

Hobo light and temperature data statistical analyses were performed separately for two time periods (Period 1: initiation through week 5; Period 2: weeks five through nine). The two time periods record light and temperature conditions in the mesocosms before and after macro- and filamentous algae removal at the end of week five from the mesocosms. To simplify the continuous recording, the data were binned into three-hour intervals: for temperature, every three hours beginning with 12 am until 9 pm, and for the light data, every three hours from 6 am until 6 pm. Each comparison was done using a paired t-test, with each difference based on the means across mesocosms for the given day/time period. The paired t-tests were performed by calculating differences between the tank means for each measurement collection time and testing whether the mean difference was significantly different from 0; this approach was used to mitigate the impact of day-to-day variability masking any impact of temperature or oysters. Due to the non-independence of consecutive days, the t-tests were performed using an “adjusted sample size” which is an adjustment to account for the fact that a calculated standard deviation of correlated values will be an underestimate of the actual variability [37]. This adjustment was done by calculating the standard error as the standard deviation divided by the square root of the adjusted sample size rather than the actual sample size. The adjusted sample size was calculated based on the autocorrelation under a presumed order 1 covariance structure, i.e., AR(1), where the difference between oyster and non-oyster mesocosms on one day was assumed to be correlated with the difference between oyster and non-oyster mesocosms for the following day [38]. All statistical analyses were conducted using SAS Version 9.4 software.

## Results

**Fig 2:**
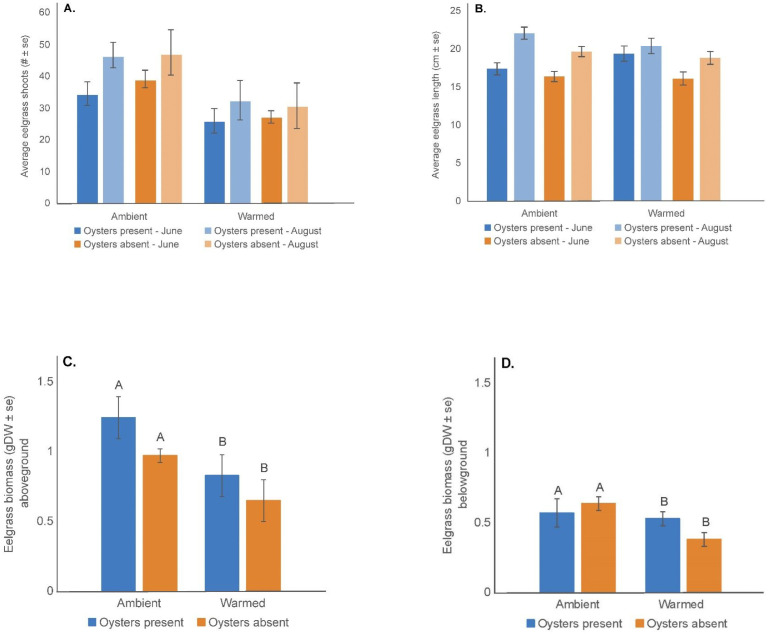
Eelgrass shoot number, length, above- and belowground biomass. (A). Mean (± se) number of eelgrass shoots recorded in the ambient and warmed mesocosms with oysters present and absent; (B). Mean (± se) eelgrass shoot length (cm) measured in the ambient and warmed mesocosms with oysters present and absent; (C). Mean (± se) eelgrass aboveground dry biomass (DW g) measured in the ambient and warmed mesocosms with oysters present and absent; (D). Mean (± se) eelgrass belowground dry biomass (DW g) measured in the ambient and warmed mesocosms with oysters present and absent. Treatments with different upper- and lower-case letters between ambient and warmed water temperatures had significantly different values (p < 0.05).

The number of live eelgrass shoots was significantly greater (F = 12.74, p = 0.0024) in the ambient mesocosms than in the warmed mesocosms with no effect of month or the presence of oysters (Fig 2a, S1 Table). While there was no difference in the live shoot lengths among treatments, as expected, the shoot lengths at the end of the experiment in August, (mean shoot length: 21.20 cm ± 0.45) were significantly greater (F = 13.50, p = 0.002) than shoot lengths in June measured two weeks post initiation (mean shoot length: 17.20 cm ± 0.41) (Fig 2b, S2a and S2b Tables). Under ambient water temperatures, the aboveground (F = 8.11, p = 0.0150) and belowground eelgrass biomass (F = 6.23, p = 0.0281) in August were significantly greater than the warmed conditions, but there was no apparent oyster effect on either measurement (Fig 2c and 2d, S3 and S4 Tables).

There were no significant differences in the initial average live wet weight of oysters between treatments in June (66.3 g ± 19.2) or at the end of the experiment in August (56.6 g ± 26.7) (S5a and S5b Tables); however, there was a loss of ~15.6% live soft tissue weight. Dry weight of the soft tissue from a subsample of five oysters from each of the eight mesocosms containing oysters (n = 40) in August demonstrated a significant temperature effect with ambient values greater than warmed water values (F = 5.56, p = 0.0462, S6 Table). The dry weight of the epiphytes on the oyster shells in the warmed seawater mesocosms (13.7 ± 2.8 mg) in July was nearly twice as great (F = 7.70, p = 0.0322, S7a Table) as in the ambient seawater mesocosms (7.6 ± 0.7 mg). In August there was no difference in epiphyte cover on the oyster shells among temperature treatments (F = 0.12, p = 0.7365, S7b Table).

**Fig 3:**
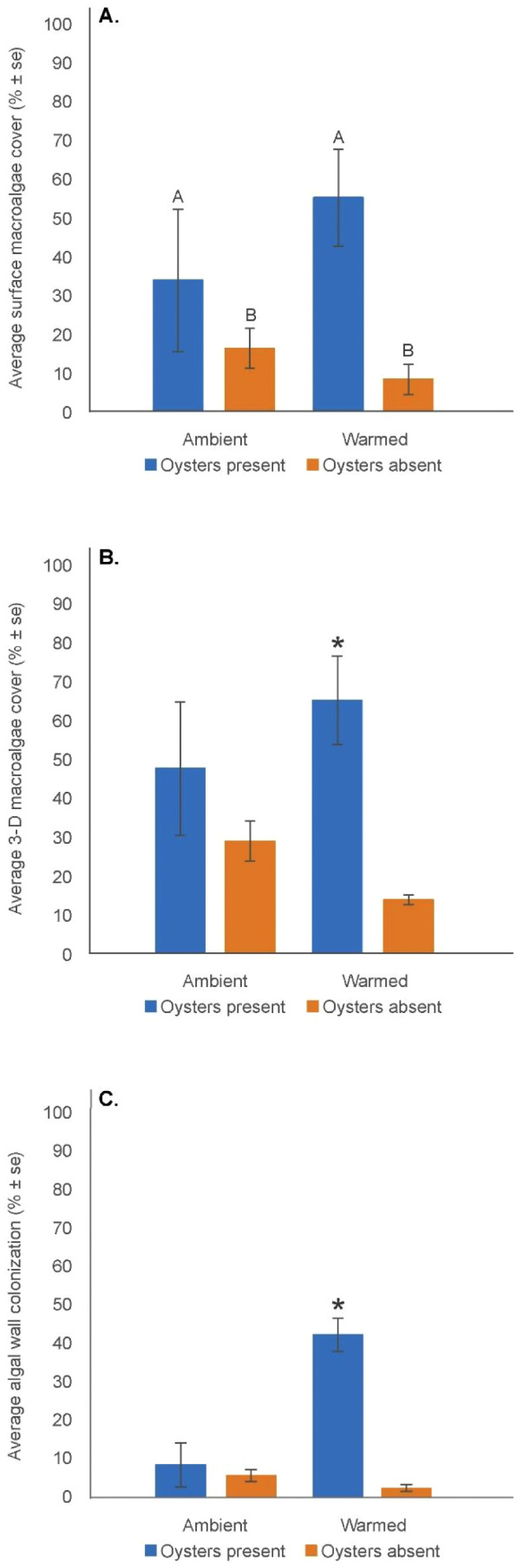
Macroalgae. (A). Mean (± se) % surface macroalgae cover; (B). Mean (± se) % 3 – D macroalgal cover; (C). Mean (± se) % algal wall colonization. Significant (p < 0.05) oyster effect on the % surface macroalgae indicated by different upper-case letters. Significant (p < 0.05) synergistic interaction effect of oyster presence and warmed water conditions indicated by an asterisk.

A variety of filamentous and macroalgae were observed across treatments, including colonial diatoms, tubular *Ulva* species, *Ceramimum, Gracilaria,* and *Cladophora* species, and other filamentous red algae. At the end of the experiment, mean percent surface macro- and filamentous algae was three times greater (F = 7.81, p = 0.0160) in mesocosms in the presence of oysters (44.4%) than in the absence of oysters (12.3%), across both ambient and warmed treatments (Fig 3a, S8 Table). In addition, percent 3 – D cover estimates of macro- and filamentous algae in the entire mesocosm showed a significant oyster by temperature interaction (F = 5.65, p = 0.035) with greater mean algal cover in the presence of oysters in the warmed mesocosms (65.0 ± 11.38%), but no effect in the presence or absence of oysters in the ambient mesocosms or the absence of oysters in the warmed mesocosms (Fig 3b, S9 Table). The presence of oysters yielded a significantly greater mean percent algal cover on the mesocosm walls in warmed treatments (42.5 ± 4.3%), but the presence or absence of oysters in ambient treatments or the absence of oysters in the warmed treatment had no effect on the percent algal cover on the mesocosm walls (F = 12.84, p = 0.004) (Fig 3c, S10 Table).

**Fig 4:**
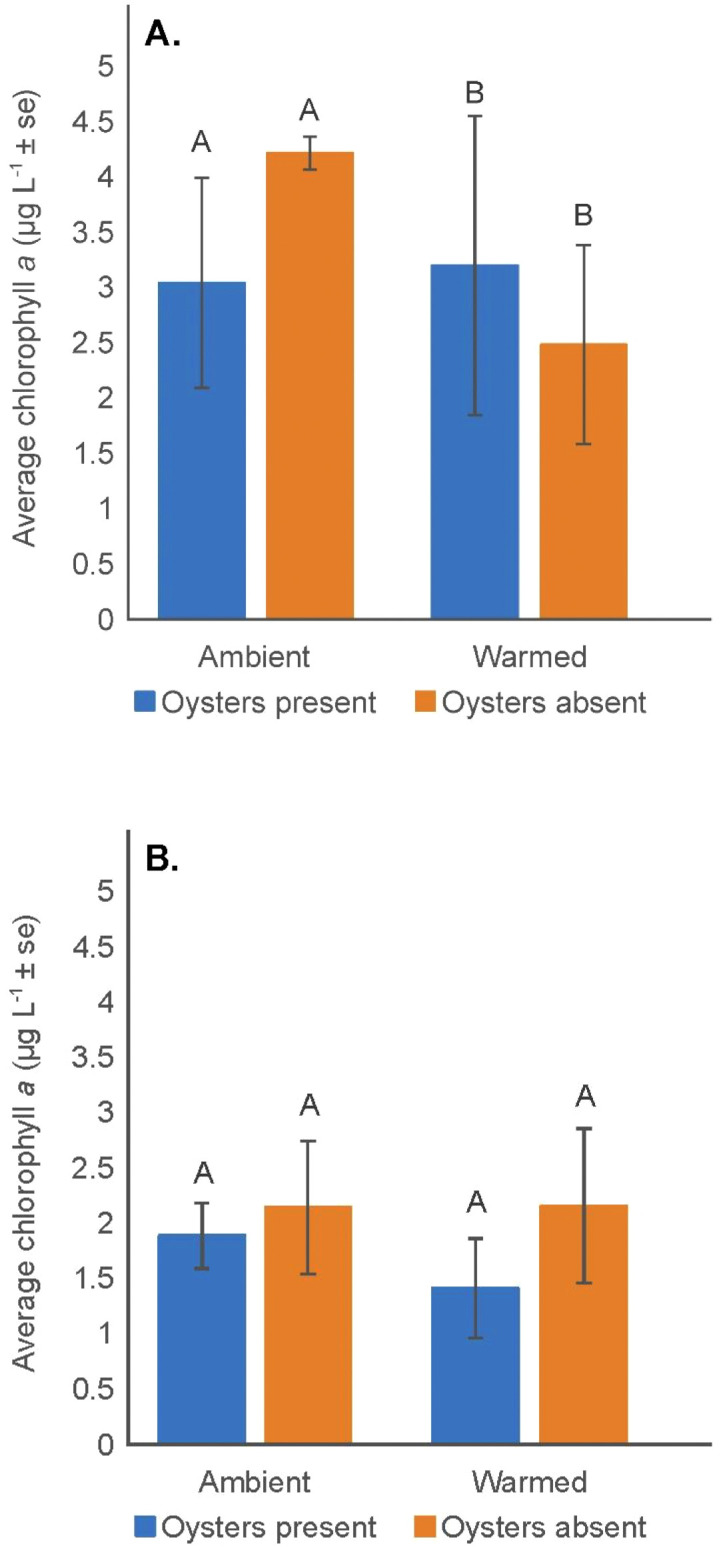
Chlorophyll *a* concentration. (A). Mean (± se) chlorophyll *a* concentration (µgL^-1^) measured at high tide (HT) and (B). Mean (± se) chlorophyll *a* concentration (µgL^-1^) measured at low tide (LT), in the ambient and warmed mesocosms with oysters present and absent. Treatments with different upper and/or lower-case letters between ambient and warmed water temperature had significantly different values (p < 0.05).

Average chlorophyll *a* concentrations in both ambient and warmed temperatures with oysters present and absent were low (< 3.0 µg L^-1^) during the June measurement and increased by up to two times during the August sampling. There was not, however, a significant interaction between chlorophyll *a* and sampling month, oyster presence, or temperature. No significant overall temperature or oyster effects on chlorophyll *a* concentrations were observed during low tide periods; however, temperature did impact chlorophyll *a* concentrations during high tide (F = 4.51, p = 0.0437), with concentrations significantly lower in warmed tanks (Fig 4a, S11a Table). Unexpectedly, there was no main effect of the presence of the filter feeding oysters on chlorophyll *a* concentrations at high and low tide (Fig 4a and 4b, S11a and S11b Tables).

Significant interactions did not occur between temperature, oysters, and month for DOC for either high or low tide. However, temperature did significantly impact DOC at high tide (F = 7.80, p = 0.0099) with higher DOC observed in warmed mesocosms. Temperature did not significantly impact DOC at low tide, and oysters did not significantly impact DOC at either tide (Table 1, S12a and S12b Tables). There were significant interactions found between month and temperature for DIC at both high (F = 11.94, p = 0.0020) and at low (F = 9.77, p = 0.0044) tide. Specifically, warmed mesocosms had significantly higher DIC than ambient mesocosms in August but not June during both high and low tides. The presence of oysters did not impact DIC significantly for either tide (Table 1, S13a and S13b Tables).

Under high and low tidal conditions, there was no temperature by oyster interaction on NH_3_, NO_2_^-^, NO_x_^-^, and PO_4_^3-^ concentrations (Table 1) (S14–S17 Tables). However, the presence of oysters did interact significantly with sampling month under high tidal conditions for NH_3_ (F = 4.93, p = 0.0358), NO_2-_ (F = 9.09, p = 0.0058) and PO_4_^3-^ (F = 9.75, p = 0.0045). Nitrite concentration was significantly greater when oysters were present in August, but there was no difference in June. For NH_3_ and PO_4_^3-^, while the presence of oysters resulted in differences of varying directions across the two months (lower concentration for oyster mesocosms in June, higher concentration in August), the magnitude of the month-specific differences was not large enough to result in statistical significance when evaluated as pairwise comparisons. No statistically significant impacts of temperature were observed for any nutrient, and no interactions or main effects of any variable were observed for NO_3-_, during the high tidal periods. During low tidal conditions, temperature and sampling month interacted significantly for NO_2-_ (F = 6.51, p = 0.0172) and PO_4_^3-^ (F = 19.08, p = 0.0002). For both parameters, the concentrations were significantly greater for ambient mesocosms during June, but there was no impact of temperature in August. No significant interactions among study variables occurred for NH_3_ or NO_3-_, and no significant impact of oyster presence was observed for any nutrients, during low tidal conditions.

As expected, water temperatures measured in the warmed mesocosms during June (2 weeks post-initiation), July (5 weeks post-initiation; low tide only), and August (9 weeks post-initiation) using the YSI were significantly elevated at high (ambient: 22.41 °C, warmed: 23.99 °C; F = 180.67, p < 0.001) tidal conditions. For low tide, temperature interacted significantly with month (F = 5.44, p = 0.007), with higher temperature occurring for warmed conditions in August only (ambient: 24.15 °C, warmed: 25.91 °C). At high tide, there was a minor, but significant cooling effect (F = 8.54, p = 0.007) of oysters on water temperatures (presence: 23.03 °C, absence: 23.37 °C) (S18a and S18b Tables). This cooling effect of oysters and associated temperature differentials were more closely tracked by the Hobo pendants in each mesocosm.

There was no temperature by oyster interaction on percent DO at either high or low tide conditions (Table 1); however, there was a significant month-by-temperature interaction at low tidal conditions (F = 4.86, p = 0.0115), with DO significantly greater in ambient tanks in August, but with no difference in June or July. The presence of oysters did not significantly impact DO in either tidal period (Table 1, S19a and S19b Tables).

Daily records of water temperature collected with the Hobo pendants showed significant cooling effects of oysters in the warmed mesocosms during some times of the day in both periods. During Period 1 at 12 am and at the evaluated time periods from 12 pm through 9 pm there was a significant cooling effect associated with the presence of oysters; while in Period 2, significant cooling effects occurred at 9 pm and at the evaluated time periods from 12 am through 12 pm (Table 2). In the ambient mesocosms in Periods 1 and 2, only at 3 pm was a cooling effect observed (Table 2). In contrast, in Period 2 in the ambient plus oyster mesocosms, there was a significant warming effect during 9 am and 12 pm (Table 2).

**Table 2:** Mean differences in temperature (oyster presence minus oyster absence) in warmed versus ambient mesocosm treatments. A positive mean value indicates a positive impact of oyster presence on temperature and a negative mean value indicates a negative impact of oyster presence on temperature. Paired t-tests were used for analyses with each difference based on means across mesocosms for the given day/time. Due to autocorrelation, tests were adjusted by determining effective sample size based on AR (1) structure.

Time Period (2021)	Time of Day	Tide Cycle	Warmed Tanks			Ambient Tanks		
			^a^Mean Difference	t statistic	p value	^a^Mean Difference	t statistic	p value
**Period 1 (6/9–6/30);** **22 days**	12:00 AM	Flood	−0.30	−4.72	0.0001	−0.21	−1.85	ns
	3:00 AM	High	−0.25	−1.63	ns	−0.15	−1.97	ns
	6:00 AM	Ebb	−0.27	−1.30	ns	−0.10	−0.94	ns
	9:00 AM	Low	0.11	0.80	ns	0.00	−0.02	ns
	12:00 PM	Flood	−0.13	−2.87	0.0094	−0.12	−0.87	ns
	3:00 PM	High	−0.27	−3.28	0.0038	−0.57	−5.18	0.0000
	6:00 PM	Ebb	−0.23	−2.92	0.0084	−0.29	−1.74	ns
	9:00 PM	Low	−0.29	−2.67	0.0146	−0.16	−1.00	ns
**Period 2 (7/5–8/4); 30 days**	12:00 AM	Flood	−0.38	−6.97	0.0000	0.16	1.88	ns
	3:00 AM	High	−0.21	−4.31	0.0002	0.14	1.77	ns
	6:00 AM	Ebb	−0.25	−6.00	0.0000	0.16	1.60	ns
	9:00 AM	Low	−0.16	−3.00	0.0055	0.23	2.63	0.0135
	12:00 PM	Flood	−0.31	−2.08	0.0465	0.17	2.35	0.0256
	3:00 PM	High	−0.31	−2.00	ns	−0.16	−2.51	0.0180
	6:00 PM	Ebb	−0.23	−1.59	ns	−0.05	−0.95	ns
	9:00 PM	Low	−0.32	−4.56	0.0001	0.08	1.19	ns

^a^Mean difference is calculated as oyster presence minus oyster absence temperature (°C).

In the morning (6 am, 9 am, and 12 pm) there were significant positive effects of oysters on light under both ambient and warmed conditions in Period 1, but significant positive effects of oysters on light were only detected for the ambient mesocosms at 9 am, 12 pm and 6 pm in Period 2 (Table 3). In contrast, in Period 1, from 3 pm and 6 pm there was a significant negative effect of oysters on light in the warmed mesocosms, but the negative effect was only detected at 3 pm in the ambient mesocosms (Table 3).

**Table 3:** Differences in light in warmed versus ambient mesocosms calculated as oyster presence minus oyster absence. Positive mean values indicate positive impact of oyster presence, negative mean values indicate negative impact of oyster presence. Analyses compared using paired t-tests with each difference based on means across mesocosms for the given day/time. Due to autocorrelation, tests were adjusted by determining effective sample size based on AR (1) structure.

Time Period (2021)	Time of Day	Tide Cycle	Warmed Tanks			Ambient Tanks		
			^a^Mean Difference	t statistic	p value	^a^Mean Difference	t statistic	p value
**Period 1 (6/9–6/30); 22 days**	6:00 AM	Ebb	0.17	4.72	0.0001	0.28	7.06	0.0000
	9:00 AM	Low	0.53	6.33	0.0000	0.47	8.31	0.0000
	12:00 PM	Flood	0.20	2.94	0.0081	0.35	6.80	0.0000
	3:00 PM	High	−0.19	−3.75	0.0013	−0.32	−3.08	0.0059
	6:00 PM	Ebb	−0.04	−2.30	0.0322	0.14	1.65	ns
**Period 2 (7/5–8/4); 30 days**	6:00 AM	Ebb	−0.19	−0.66	ns	0.18	2.02	ns
	9:00 AM	Low	0.07	0.71	ns	0.32	3.81	0.0007
	12:00 PM	Flood	−0.30	−1.02	ns	0.28	4.49	0.0001
	3:00 PM	High	−0.40	−1.44	ns	−0.21	−1.73	ns
	6:00 PM	Ebb	−0.39	−1.68	ns	0.11	3.19	0.0034

^a^Mean difference calculated as oyster presence minus oyster absence natural log light.

## Discussion

**Fig 5:**
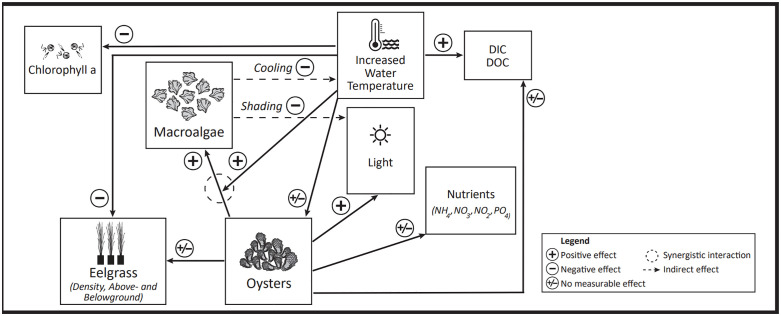
Diagrammatic representation of the interactions between oysters and seagrass.

This nine-week mesocosm experiment was designed to examine the effects of oysters, water temperature, and their interactions on eelgrass growth, macroalgae cover, chlorophyll *a*, dissolved nutrient concentrations, and water quality variables. Our results partially supported our hypotheses. As hypothesized, under warmed water temperatures, eelgrass shoot number, and above- and belowground biomass declined, as did chlorophyll *a* concentrations during the high tide cycle. Oyster biomass did decline during the nine-week period indicating insufficient phytoplankton resources, although the oysters were observed to be actively feeding. Contrary to our hypothesis, the presence of oysters in the mesocosms did not affect chlorophyll *a* concentrations due to their filtering processes. Oysters did have some positive effects on light availability for some parts of the day, especially in the morning (ebbing and low tides) in both the ambient and warmed temperatures, however, during the afternoon (high tide) there were often negative effects of oysters on light availability. Under natural conditions these contrasting effects of oysters on light availability might be related to the movement of drifting macroalgae and associated shading, which may sometimes offset the positive effects of oyster filtration. Shading in the mesocosms and the greenhouse as the angle of light changed during the day might mimic the drifting macroalgae shading in the wild. Oysters increased the percent cover of surface macroalgae at both warmed and ambient temperatures, and the presence of oysters and warmed conditions had a synergistic effect on the 3 – D % cover and algal wall colonization in the mesocosms (Fig 5).

Oysters excrete nitrogen predominantly in the form of NH_3_ [39] with higher rates at warmer temperatures [40]. This biologically available NH_3_ may be readily converted into increased productivity of algal species [16] which was evident, particularly in the enclosed mesocosm experimental system and warmed seawater. The finding of no differences in ammonia concentrations between mesocosms with oysters present or absent may be explained as the nitrogenous waste from oyster excretion was bound in the algal biomass. Elevated DIC concentrations in the warmed mesocosms at both tidal stages in August may have resulted from the degradation of plankton by bacteria with respiration outpacing photosynthesis, increasing CO_2_ concentrations. Additionally, there is the potential that eelgrass was responsible for the observed changes in DIC and DOC measures across the treatments. At the warmer temperatures, eelgrass may take up less DIC and through decomposition of the plant material may be generating greater DOC. These DIC findings suggest the biological activity in the warmed mesocosms mirrors the seasonal reduction in DIC concentrations in the summer months reported from sampling stations in the southern portions of Narragansett Bay, the source water for the mesocosms [41]. The DOC concentrations were greater at the warmed temperatures at high tide which suggests the warmer temperatures facilitated a greater breakdown of organic matter (i.e., eelgrass and algae) than in the ambient temperature mesocosms.

Interactions between oysters and seagrass have been described as reciprocal with both positive and negative attributes which are generally spatially and temporally specific to a geographic setting [42]. Suspension feeding bivalves act as a pelagic-benthic conduit clearing particles from the water column and depositing their nutrient rich biodeposits on the sediment surface, improving light for seagrass and enhancing plant productivity [19], however the presence of shellfish (i.e., mussels) may also negatively impact seagrass productivity through increased concentrations of sediment sulphide [43]. Seagrasses have a role in improving bivalve survival by concentrating food particles for the oysters and increasing their growth rates [19,42]. Oysters which were co-cultured with eelgrass in mesocosms enhanced eelgrass above and belowground growth metrics at ambient water temperatures, while at increased temperature the presence of oysters negatively impacted some of these metrics [44]. We did not observe an oyster effect on either chlorophyll *a* concentrations or eelgrass growth metrics which may be due to the short-term nature of the experiment. In addition, the oysters were not in direct contact with the eelgrass sediments so direct benefits from their nutrient rich biodeposits may not have been realized. However, there may have been an indirect effect of oysters on the eelgrass system mediated by the increased cover of macroalgae. A significant cooling effect of oysters in warmed mesocosms was intermittent and recorded for some parts of the day. The patterns in daily measures of temperature and light, collected by the Hobo pendants, were not only affected by the oyster and temperature treatments, but also by the rise and fall of the tides and the macroalgae in the mesocosms.

We propose that a possible outcome of the increased macroalgae in the presence of oysters was amelioration of warming, possibly by shading, especially in the warmed mesocosms, where there was the greatest cover of macroalgae. The indirect cooling effect of oysters in the ambient mesocosms was observed less frequently than in the warmed mesocosms, and sometimes the oyster presence in the ambient mesocosms caused a slight warming effect. These novel mesocosm findings suggest the interaction of oysters and macroalgae may play a role in mitigating warming in some coastal, shallow habitats. While intermittent shading of light by macroalgae might hinder eelgrass growth [45–47], in a subtropical *Thalassia* meadow study, it was observed that moderate cover of drift macroalgae may benefit seagrass by reducing epiphyte leaf cover [48]. Reductions by approximately 50% in the canopy cover of emersed *Ascophyllum* sp. along a rocky coast increased the underlying temperature and reduced the abundance of the invertebrate community [49]. Additionally, the creation of cool microclimates by habitat forming emersed rock oyster reefs ameliorated thermal stress enabling the persistence of a diverse and abundant invertebrate fauna within the intertidal zone, as compared to bare rocky shoreline, in Hong Kong [50]. Clearly, further study is needed to determine if actual macroalgal benefits such as water temperature cooling (present study) and reduced epiphytic loads [48] occur with varying spatial and temporal patterns of algal cover, and if oyster-macroalgae interactions may play a role in cooling episodes in shallow waters under natural conditions. In the present mesocosm study we were able to examine the main effects and interactions of increasing water temperatures and the presence of oysters on seagrass systems in a controlled environment. However, field studies are essential to accompany mesocosm experimentation to better account for the complexity of environmental variables under natural conditions.

Around the continental US when water temperatures have exceeded thermal tolerances or there have been summertime heat waves, *Z. marina* die backs have occurred. Along the Atlantic coast eelgrass losses due to increased water temperatures have been reported from the Peconic Bay, New York [51,52], the Chesapeake Bay, and the York River, Virginia [53–56], and in estuaries along the Pacific northwest [57]. Our study results support these findings as the warmed water did adversely affect the eelgrass shoot density and above- and below-ground biomass regardless of the presence or absence of oysters. The results from the short-term mesocosm study help to illuminate the potential interactions between oysters, eelgrass, and warming temperatures on the ecology of northeastern U S coastal systems. We suggest the need for field studies to further explore these dynamics with the aim to improve eelgrass restoration outcomes.

## Supporting information

S1 Table Combined June and August measurement of (log) number of live shoots. Full model results from the GLM procedure.
(DOCX)

S2a Table June measurement of (log) live shoot length, including nested mesocosm factor. Full model results from the GLM procedure.
(DOCX)

S2b Table Combined June and August measurement of (log) shoot length. Full model results from the GLM procedure.
(DOCX)

S3 Table August (log) aboveground biomass of live eelgrass, at the end of the experiment. Full model results from the GLM procedure.
(DOCX)

S4 Table August (log) belowground biomass of live eelgrass, at the end of the experiment. Full model results from the GLM procedure.
(DOCX)

S5a Table Initial oyster (log) wet weight biomass. Full model results from the GLM procedure.
(DOCX)

S5b Table Final oyster (log) wet weight biomass. Full model results from the GLM procedure.
(DOCX)

S6 Table Oyster tissue/oyster shell log dry weight in August. Full model results from the GLM procedure.
(DOCX)

S7a Table Oyster shell epiphyte (log) dry weight in July. Full model results from the GLM procedure.
(DOCX)

S7b Table Oyster shell epiphyte (log) dry weight in August. Full model results from the GLM procedure.
(DOCX)

S8 Table Macroalgae (log) percent surface cover. Full model results from the GLM procedure.
(DOCX)

S9 Table Macroalgae (log) percent 3 – D cover. Full model results from the GLM procedure.
(DOCX)

S10 Table Macroalgae (log) wall colonization. Full model results from the GLM procedure.
(DOCX)

S11a Table (Log) chlorophyll *a* concentration at high tide across months. Full model results from the GLM procedure.
(DOCX)

S11b Table (Log) chlorophyll *a* concentration at low tide across months. Full model results from the GLM procedure.
(DOCX)

S12a Table Dissolved organic carbon (DOC) concentrations at high tide across months. Full model results from the GLM procedure.
(DOCX)

S12b Table Dissolved organic carbon (DOC) concentrations at low tide across months. Full model results from the GLM procedure.
(DOCX)

S13a Table Dissolved inorganic carbon (DIC) concentrations at high tide across months. Full model results from the GLM procedure.

S13b Table Dissolved inorganic carbon (DIC) concentrations at low tide across months. Full model results from the GLM procedure.
(DOCX)

S14a Table (Log) ammonia (NH_3_) concentration at high tide across months. Full model results from the GLM procedure.
(DOCX)

S14b Table (Log) ammonia (NH_3_) concentration at low tide across months. Full model results from the GLM procedure.
(DOCX)

S15a Table (Log) nitrite (NO_2_) concentration at high tide across months. Full model results from the GLM procedure.
(DOCX)

S15b Table (Log) nitrite (NO_2_) concentration at low tide across months. Full model results from the GLM procedure.
(DOCX)

S16a Table (Log) nitrate and nitrite (NO_x_) concentration at high tide across months. Full model results from the GLM procedure.
(DOCX)

S16b Table (Log) nitrate and nitrite (NO_x_^-^) concentration at low tide across months. Full model results from the GLM procedure.
(DOCX)

S17a Table (Log) orthophosphate (PO_4_^3^) concentration at high tide across months. Full model results from the GLM procedure.
(DOCX)

S17b Table (Log) orthophosphate (PO_4_^3^) concentration at low tide across months. Full model results from the GLM procedure.
(DOCX)

S18a Table Water temperature at high tide across months. Full model results from the GLM procedure.
(DOCX)

S18b Table Water temperature at low tide across months. Full model results from the GLM procedure.
(DOCX)

S19a Table Dissolved oxygen (DO) at high tide across months. Full model results from the GLM procedure.
(DOCX)

S19b Table Dissolved oxygen (DO) at low tide across months. Full model results from the GLM procedure.
(DOCX)
