# Supplementary material for: Seagrass and oyster interactions under a warming climate scenario: A mesocosm experiment
Source: PLoS One. 2025 Dec 11;20(12):e0337843. doi: 10.1371/journal.pone.0337843 (PMC12698006; doi:10.1371/journal.pone.0337843)
Supplement: S1 Table — Full model results from the GLM procedure. (DOCX) [file pone.0337843.s001.docx]

Supporting Information

S1 Table. Combined June and August measurement of (log) number of live shoots. Full model results from the GLM procedure.

Dependent Variable: (log) number of live eelgrass shoots.

| Source | DF | Sum of Squares | Mean Square | F Value | Pr > F |
| --- | --- | --- | --- | --- | --- |
| Model | 6 | 1.74948293 | 0.29158049 | 2.87 | 0.0286 |
| Error | 25 | 2.53557569 | 0.10142303 |  |  |
| Corrected Total | 31 | 4.28505862 |  |  |  |

| R-Square | Coeff Var | Root MSE | llive Mean |
| --- | --- | --- | --- |
| 0.408275 | 9.096121 | 0.318470 | 3.501161 |

| Source | DF | Type I SS | Mean Square | F Value | Pr > F |
| --- | --- | --- | --- | --- | --- |
| AmbTemp | 1 | 1.29243859 | 1.29243859 | 12.74 | 0.0015 |
| Oysters | 1 | 0.00189827 | 0.00189827 | 0.02 | 0.8923 |
| AmbTemp*Oysters | 1 | 0.05146477 | 0.05146477 | 0.51 | 0.4829 |
| month | 1 | 0.37208083 | 0.37208083 | 3.67 | 0.0670 |
| AmbTemp*month | 1 | 0.01394478 | 0.01394478 | 0.14 | 0.7139 |
| Oysters*month | 1 | 0.01765570 | 0.01765570 | 0.17 | 0.6801 |

| Source | DF | Type III SS | Mean Square | F Value | Pr > F |
| --- | --- | --- | --- | --- | --- |
| AmbTemp | 1 | 1.29243859 | 1.29243859 | 12.74 | 0.0015 |
| Oysters | 1 | 0.00189827 | 0.00189827 | 0.02 | 0.8923 |
| AmbTemp*Oysters | 1 | 0.05146477 | 0.05146477 | 0.51 | 0.4829 |
| month | 1 | 0.37208083 | 0.37208083 | 3.67 | 0.0670 |
| AmbTemp*month | 1 | 0.01394478 | 0.01394478 | 0.14 | 0.7139 |
| Oysters*month | 1 | 0.01765570 | 0.01765570 | 0.17 | 0.6801 |
