# Supplementary material for: Seagrass and oyster interactions under a warming climate scenario: A mesocosm experiment
Source: PLoS One. 2025 Dec 11;20(12):e0337843. doi: 10.1371/journal.pone.0337843 (PMC12698006; doi:10.1371/journal.pone.0337843)
Supplement: S2a Table — Full model results from the GLM procedure. (DOCX) [file pone.0337843.s002.docx]

Supporting Information

S2a Table. June measurement of (log) live shoot length, including nested mesocosm factor. Full model results from the GLM procedure.

Dependent Variable: (log) live eelgrass shoot length.

| Tests of Hypotheses Using the Type III MS for Tank(AmbTem*Oysters) as an Error Term | | | | | |
| --- | --- | --- | --- | --- | --- |
| Source | DF | Type III SS | Mean Square | F Value | Pr > F |
| AmbTemp*Oysters | 1 | 0.17839699 | 0.17839699 | 0.23 | 0.6430 |
| AmbTemp | 1 | 0.38293113 | 0.38293113 | 0.49 | 0.4993 |
| Oysters | 1 | 0.32410333 | 0.32410333 | 0.41 | 0.5337 |
