# Supplementary material for: Seagrass and oyster interactions under a warming climate scenario: A mesocosm experiment
Source: PLoS One. 2025 Dec 11;20(12):e0337843. doi: 10.1371/journal.pone.0337843 (PMC12698006; doi:10.1371/journal.pone.0337843)
Supplement: S2b Table — Full model results from the GLM procedure. (DOCX) [file pone.0337843.s003.docx]

Supporting Information

S2b Table. Combined June and August measurement of (log) shoot length. Full model results from the GLM procedure.

Dependent Variable: (log) live eelgrass shoot length.

| Source | DF | Sum of Squares | Mean Square | F Value | Pr > F |
| --- | --- | --- | --- | --- | --- |
| Model | 31 | 46.4164769 | 1.4973057 | 4.46 | <.0001 |
| Error | 1095 | 367.6801295 | 0.3357809 |  |  |
| Corrected Total | 1126 | 414.0966065 |  |  |  |

| R-Square | Coeff Var | Root MSE | llen Mean |
| --- | --- | --- | --- |
| 0.112091 | 20.91558 | 0.579466 | 2.770500 |

| Tests of Hypotheses Using the Type III MS for Tank(AmbTem*Oysters) as an Error Term | | | | | |
| --- | --- | --- | --- | --- | --- |
| Source | DF | Type III SS | Mean Square | F Value | Pr > F |
| AmbTemp*Oysters | 1 | 0.00210764 | 0.00210764 | 0.00 | 0.9771 |
| AmbTemp | 1 | 0.06851654 | 0.06851654 | 0.03 | 0.8703 |
| Oysters | 1 | 0.69854008 | 0.69854008 | 0.28 | 0.6040 |

| Tests of Hypotheses Using the Type III MS for mont*Tank(AmbT*Oyst) as an Error Term | | | | | |
| --- | --- | --- | --- | --- | --- |
| Source | DF | Type III SS | Mean Square | F Value | Pr > F |
| month | 1 | 4.77679356 | 4.77679356 | 13.50 | 0.0023 |
