# Supplementary material for: Seagrass and oyster interactions under a warming climate scenario: A mesocosm experiment
Source: PLoS One. 2025 Dec 11;20(12):e0337843. doi: 10.1371/journal.pone.0337843 (PMC12698006; doi:10.1371/journal.pone.0337843)
Supplement: S3 Table — Full model results from the GLM procedure. (DOCX) [file pone.0337843.s004.docx]

**Supporting Information**

**S3 Table. August (log) aboveground biomass of live eelgrass, at the end of the experiment. Full model results from the GLM procedure.**

Dependent variable: (log) aboveground biomass of live eelgrass.

| Source | DF | Sum of Squares | Mean Square | F Value | Pr > F |
| --- | --- | --- | --- | --- | --- |
| Model | 3 | 1.07063666 | 0.35687889 | 3.62 | 0.0456 |
| Error | 12 | 1.18464663 | 0.09872055 |  |  |
| Corrected Total | 15 | 2.25528329 |  |  |  |

| R-Square | Coeff Var | Root MSE | lla Mean |
| --- | --- | --- | --- |
| 0.474724 | -219.1613 | 0.314198 | -0.143364 |

| Source | DF | Type I SS | Mean Square | F Value | Pr > F |
| --- | --- | --- | --- | --- | --- |
| AmbTemp | 1 | 0.80040233 | 0.80040233 | 8.11 | 0.0147 |
| Oysters | 1 | 0.26594747 | 0.26594747 | 2.69 | 0.1267 |
| AmbTemp*Oysters | 1 | 0.00428685 | 0.00428685 | 0.04 | 0.8384 |

| Source | DF | Type III SS | Mean Square | F Value | Pr > F |
| --- | --- | --- | --- | --- | --- |
| AmbTemp | 1 | 0.80040233 | 0.80040233 | 8.11 | 0.0147 |
| Oysters | 1 | 0.26594747 | 0.26594747 | 2.69 | 0.1267 |
| AmbTemp*Oysters | 1 | 0.00428685 | 0.00428685 | 0.04 | 0.8384 |
