# Supplementary material for: Seagrass and oyster interactions under a warming climate scenario: A mesocosm experiment
Source: PLoS One. 2025 Dec 11;20(12):e0337843. doi: 10.1371/journal.pone.0337843 (PMC12698006; doi:10.1371/journal.pone.0337843)
Supplement: S4 Table — Full model results from the GLM procedure. (DOCX) [file pone.0337843.s005.docx]

Supporting Information

S4 Table. August (log) belowground biomass of live eelgrass, at the end of the experiment. Full model results from the GLM procedure.

Dependent variable: (log) belowground biomass of live eelgrass.

| Source | DF | Sum of Squares | Mean Square | F Value | Pr > F |
| --- | --- | --- | --- | --- | --- |
| Model | 3 | 0.65426877 | 0.21808959 | 3.74 | 0.0418 |
| Error | 12 | 0.70062339 | 0.05838528 |  |  |
| Corrected Total | 15 | 1.35489216 |  |  |  |

| R-Square | Coeff Var | Root MSE | lbg Mean |
| --- | --- | --- | --- |
| 0.482894 | -35.83442 | 0.241630 | -0.674297 |

| Source | DF | Type I SS | Mean Square | F Value | Pr > F |
| --- | --- | --- | --- | --- | --- |
| AmbTemp | 1 | 0.36381475 | 0.36381475 | 6.23 | 0.0281 |
| Oysters | 1 | 0.04902007 | 0.04902007 | 0.84 | 0.3776 |
| AmbTemp*Oysters | 1 | 0.24143395 | 0.24143395 | 4.14 | 0.0647 |

| Source | DF | Type III SS | Mean Square | F Value | Pr > F |
| --- | --- | --- | --- | --- | --- |
| AmbTemp | 1 | 0.36381475 | 0.36381475 | 6.23 | 0.0281 |
| Oysters | 1 | 0.04902007 | 0.04902007 | 0.84 | 0.3776 |
| AmbTemp*Oysters | 1 | 0.24143395 | 0.24143395 | 4.14 | 0.0647 |
