# Supplementary material for: Seagrass and oyster interactions under a warming climate scenario: A mesocosm experiment
Source: PLoS One. 2025 Dec 11;20(12):e0337843. doi: 10.1371/journal.pone.0337843 (PMC12698006; doi:10.1371/journal.pone.0337843)
Supplement: S5a Table — Full model results from the GLM procedure. (DOCX) [file pone.0337843.s006.docx]

Supporting Information

S5a Table. Initial oyster (log) wet weight biomass. Full model results from the GLM procedure.

Dependent variable: initial oyster (log) biomass.

| Source | DF | Sum of Squares | Mean Square | F Value | Pr > F |
| --- | --- | --- | --- | --- | --- |
| Model | 3 | 0.02555582 | 0.00851861 | 0.39 | 0.7620 |
| Error | 8 | 0.17368074 | 0.02171009 |  |  |
| Corrected Total | 11 | 0.19923657 |  |  |  |

| R-Square | Coeff Var | Root MSE | lbeginwt  Mean |
| --- | --- | --- | --- |
| 0.128269 | 1.941941 | 0.147343 | 7.587434 |

| Source | DF | Type I SS | Mean Square | F Value | Pr > F |
| --- | --- | --- | --- | --- | --- |
| AmbTemp | 1 | 0.01061974 | 0.01061974 | 0.49 | 0.5041 |
| Eelgrass | 1 | 0.00268574 | 0.00268574 | 0.12 | 0.7341 |
| AmbTemp*Eelgrass | 1 | 0.01225034 | 0.01225034 | 0.56 | 0.4741 |

| Source | DF | Type III SS | Mean Square | F Value | Pr > F |
| --- | --- | --- | --- | --- | --- |
| AmbTemp | 1 | 0.01797000 | 0.01797000 | 0.83 | 0.3895 |
| Eelgrass | 1 | 0.00268574 | 0.00268574 | 0.12 | 0.7341 |
| AmbTemp*Eelgrass | 1 | 0.01225034 | 0.01225034 | 0.56 | 0.4741 |
