# Supplementary material for: Seagrass and oyster interactions under a warming climate scenario: A mesocosm experiment
Source: PLoS One. 2025 Dec 11;20(12):e0337843. doi: 10.1371/journal.pone.0337843 (PMC12698006; doi:10.1371/journal.pone.0337843)
Supplement: S5b Table — Full model results from the GLM procedure. (DOCX) [file pone.0337843.s007.docx]

Supporting Information

S5b Table. Final oyster (log) wet weight biomass. Full model results from the GLM procedure.

Dependent variable: final oyster (log) biomass.

| Source | DF | Sum of Squares | Mean Square | F Value | Pr > F |
| --- | --- | --- | --- | --- | --- |
| Model | 3 | 0.00521399 | 0.00173800 | 0.02 | 0.9943 |
| Error | 8 | 0.56003892 | 0.07000487 |  |  |
| Corrected Total | 11 | 0.56525291 |  |  |  |

| R-Square | Coeff Var | Root MSE | lendwt  Mean |
| --- | --- | --- | --- |
| 0.009224 | 3.559629 | 0.264584 | 7.432920 |

| Source | DF | Type I SS | Mean Square | F Value | Pr > F |
| --- | --- | --- | --- | --- | --- |
| AmbTemp | 1 | 0.00000688 | 0.00000688 | 0.00 | 0.9923 |
| Eelgrass | 1 | 0.00367481 | 0.00367481 | 0.05 | 0.8245 |
| AmbTemp*Eelgrass | 1 | 0.00153230 | 0.00153230 | 0.02 | 0.8860 |

| Source | DF | Type III SS | Mean Square | F Value | Pr > F |
| --- | --- | --- | --- | --- | --- |
| AmbTemp | 1 | 0.00024088 | 0.00024088 | 0.00 | 0.9547 |
| Eelgrass | 1 | 0.00367481 | 0.00367481 | 0.05 | 0.8245 |
| AmbTemp*Eelgrass | 1 | 0.00153230 | 0.00153230 | 0.02 | 0.8860 |
