# Supplementary material for: Seagrass and oyster interactions under a warming climate scenario: A mesocosm experiment
Source: PLoS One. 2025 Dec 11;20(12):e0337843. doi: 10.1371/journal.pone.0337843 (PMC12698006; doi:10.1371/journal.pone.0337843)
Supplement: S6 Table — Full model results from the GLM procedure. (DOCX) [file pone.0337843.s008.docx]

Supporting Information

S6 Table. Oyster tissue/oyster shell log dry weight in August. Full model results from the GLM procedure.

Dependent variable: oyster tissue/shell (log) dry weight.

| Source | DF | Sum of Squares | Mean Square | F Value | Pr > F |
| --- | --- | --- | --- | --- | --- |
| Model | 11 | 0.68685112 | 0.06244101 | 0.68 | 0.7488 |
| Error | 48 | 4.39781460 | 0.09162114 |  |  |
| Corrected Total | 59 | 5.08466572 |  |  |  |

| R-Square | Coeff Var | Root MSE | lasdw Mean |
| --- | --- | --- | --- |
| 0.135083 | 7.957689 | 0.302690 | 3.803740 |

| Tests of Hypotheses Using the Type III MS for tank(AmbTem*Eelgras) as an Error Term | | | | | |
| --- | --- | --- | --- | --- | --- |
| Source | DF | Type III SS | Mean Square | F Value | Pr > F |
| AmbTemp*Eelgrass | 1 | 0.10902222 | 0.10902222 | 3.19 | 0.1117 |
| AmbTemp | 1 | 0.18970718 | 0.18970718 | 5.56 | 0.0462 |
| Eelgrass | 1 | 0.17161660 | 0.17161660 | 5.03 | 0.0552 |
