# Supplementary material for: Seagrass and oyster interactions under a warming climate scenario: A mesocosm experiment
Source: PLoS One. 2025 Dec 11;20(12):e0337843. doi: 10.1371/journal.pone.0337843 (PMC12698006; doi:10.1371/journal.pone.0337843)
Supplement: S7a Table — Full model results from the GLM procedure. (DOCX) [file pone.0337843.s009.docx]

Supporting Information

S7a Table. Oyster shell epiphyte (log) dry weight in July. Full model results from the GLM procedure.

Dependent variable: Epiphyte (log) dry weight in July.

| Source | DF | Sum of Squares | Mean Square | F Value | Pr > F |
| --- | --- | --- | --- | --- | --- |
| Model | 1 | 0.59515360 | 0.59515360 | 7.70 | 0.0322 |
| Error | 6 | 0.46396775 | 0.07732796 |  |  |
| Corrected Total | 7 | 1.05912134 |  |  |  |

| R-Square | Coeff Var | Root MSE | ldw Mean |
| --- | --- | --- | --- |
| 0.561931 | 12.12813 | 0.278079 | 2.292843 |

| Source | DF | Type I SS | Mean Square | F Value | Pr > F |
| --- | --- | --- | --- | --- | --- |
| AmbTemp | 1 | 0.59515360 | 0.59515360 | 7.70 | 0.0322 |

| Source | DF | Type III SS | Mean Square | F Value | Pr > F |
| --- | --- | --- | --- | --- | --- |
| AmbTemp | 1 | 0.59515360 | 0.59515360 | 7.70 | 0.0322 |
