# Supplementary material for: Seagrass and oyster interactions under a warming climate scenario: A mesocosm experiment
Source: PLoS One. 2025 Dec 11;20(12):e0337843. doi: 10.1371/journal.pone.0337843 (PMC12698006; doi:10.1371/journal.pone.0337843)
Supplement: S7b Table — Full model results from the GLM procedure. (DOCX) [file pone.0337843.s010.docx]

**Supporting Information**

**S7b Table. Oyster shell epiphyte (log) dry weight in August. Full model results from the GLM procedure.**

Dependent variable: Epiphyte (log) dry weight in August.

| Source | DF | Sum of Squares | Mean Square | F Value | Pr > F |
| --- | --- | --- | --- | --- | --- |
| Model | 1 | 0.00003306 | 0.00003306 | 0.00 | 0.9924 |
| Error | 6 | 2.02057433 | 0.33676239 |  |  |
| Corrected Total | 7 | 2.02060739 |  |  |  |

| R-Square | Coeff Var | Root MSE | ldw Mean |
| --- | --- | --- | --- |
| 0.000016 | 45.25561 | 0.580312 | 1.282299 |

| Source | DF | Type I SS | Mean Square | F Value | Pr > F |
| --- | --- | --- | --- | --- | --- |
| AmbTemp | 1 | 0.00003306 | 0.00003306 | 0.00 | 0.9924 |

| Source | DF | Type III SS | Mean Square | F Value | Pr > F |
| --- | --- | --- | --- | --- | --- |
| AmbTemp | 1 | 0.00003306 | 0.00003306 | 0.00 | 0.9924 |
