# Supplementary material for: Seagrass and oyster interactions under a warming climate scenario: A mesocosm experiment
Source: PLoS One. 2025 Dec 11;20(12):e0337843. doi: 10.1371/journal.pone.0337843 (PMC12698006; doi:10.1371/journal.pone.0337843)
Supplement: S8 Table — Full model results from the GLM procedure. (DOCX) [file pone.0337843.s011.docx]

Supporting Information

S8 Table. Macroalgae (log) percent surface cover. Full model results from the GLM procedure.

Dependent variable: Macroalgae (log) surface percent cover.

| Source | DF | Sum of Squares | Mean Square | F Value | Pr > F |
| --- | --- | --- | --- | --- | --- |
| Model | 3 | 9.00039470 | 3.00013157 | 3.88 | 0.0376 |
| Error | 12 | 9.27078053 | 0.77256504 |  |  |
| Corrected Total | 15 | 18.27117523 |  |  |  |

| R-Square | Coeff Var | Root MSE | lsurf Mean |
| --- | --- | --- | --- |
| 0.492601 | 31.04069 | 0.878957 | 2.831627 |

| Source | DF | Type I SS | Mean Square | F Value | Pr > F |
| --- | --- | --- | --- | --- | --- |
| AmbTemp | 1 | 0.02500530 | 0.02500530 | 0.03 | 0.8602 |
| Oysters | 1 | 6.03454754 | 6.03454754 | 7.81 | 0.0162 |
| AmbTemp*Oysters | 1 | 2.94084186 | 2.94084186 | 3.81 | 0.0748 |

| Source | DF | Type III SS | Mean Square | F Value | Pr > F |
| --- | --- | --- | --- | --- | --- |
| AmbTemp | 1 | 0.02500530 | 0.02500530 | 0.03 | 0.8602 |
| Oysters | 1 | 6.03454754 | 6.03454754 | 7.81 | 0.0162 |
| AmbTemp*Oysters | 1 | 2.94084186 | 2.94084186 | 3.81 | 0.0748 |
