# Supplementary material for: Seagrass and oyster interactions under a warming climate scenario: A mesocosm experiment
Source: PLoS One. 2025 Dec 11;20(12):e0337843. doi: 10.1371/journal.pone.0337843 (PMC12698006; doi:10.1371/journal.pone.0337843)
Supplement: S9 Table — Full model results from the GLM procedure. (DOCX) [file pone.0337843.s012.docx]

Supporting Information

S9 Table. Macroalgae (log) percent 3 - D cover. Full model results from the GLM procedure.

Dependent variable: Macroalgae (log) percent 3 – D cover.

| Source | DF | Sum of Squares | Mean Square | F Value | Pr > F |
| --- | --- | --- | --- | --- | --- |
| Model | 3 | 4.82402399 | 1.60800800 | 6.41 | 0.0077 |
| Error | 12 | 3.00801339 | 0.25066778 |  |  |
| Corrected Total | 15 | 7.83203738 |  |  |  |

| R-Square | Coeff Var | Root MSE | l3d Mean |
| --- | --- | --- | --- |
| 0.615935 | 14.65603 | 0.500667 | 3.416117 |

| Source | DF | Type I SS | Mean Square | F Value | Pr > F |
| --- | --- | --- | --- | --- | --- |
| AmbTemp | 1 | 0.04576028 | 0.04576028 | 0.18 | 0.6768 |
| Oysters | 1 | 3.36082666 | 3.36082666 | 13.41 | 0.0033 |
| AmbTemp*Oysters | 1 | 1.41743706 | 1.41743706 | 5.65 | 0.0349 |

| Source | DF | Type III SS | Mean Square | F Value | Pr > F |
| --- | --- | --- | --- | --- | --- |
| AmbTemp | 1 | 0.04576028 | 0.04576028 | 0.18 | 0.6768 |
| Oysters | 1 | 3.36082666 | 3.36082666 | 13.41 | 0.0033 |
| AmbTemp*Oysters | 1 | 1.41743706 | 1.41743706 | 5.65 | 0.0349 |
