# Supplementary material for: Seagrass and oyster interactions under a warming climate scenario: A mesocosm experiment
Source: PLoS One. 2025 Dec 11;20(12):e0337843. doi: 10.1371/journal.pone.0337843 (PMC12698006; doi:10.1371/journal.pone.0337843)
Supplement: S10 Table — Full model results from the GLM procedure. (DOCX) [file pone.0337843.s013.docx]

Supporting Information

S10 Table. Macroalgae (log) wall colonization. Full model results from the GLM procedure.

Dependent variable: Macroalgae (log) wall colonization

| Source | DF | Sum of Squares | Mean Square | F Value | Pr > F |
| --- | --- | --- | --- | --- | --- |
| Model | 3 | 16.20637597 | 5.40212532 | 8.12 | 0.0032 |
| Error | 12 | 7.98492739 | 0.66541062 |  |  |
| Corrected Total | 15 | 24.19130337 |  |  |  |

| R-Square | Coeff Var | Root MSE | labove  Mean |
| --- | --- | --- | --- |
| 0.669926 | 39.41122 | 0.815727 | 2.069784 |

| Source | DF | Type I SS | Mean Square | F Value | Pr > F |
| --- | --- | --- | --- | --- | --- |
| AmbTemp | 1 | 2.31358252 | 2.31358252 | 3.48 | 0.0869 |
| Oysters | 1 | 5.35046202 | 5.35046202 | 8.04 | 0.0150 |
| AmbTemp*Oysters | 1 | 8.54233143 | 8.54233143 | 12.84 | 0.0038 |

| Source | DF | Type III SS | Mean Square | F Value | Pr > F |
| --- | --- | --- | --- | --- | --- |
| AmbTemp | 1 | 2.31358252 | 2.31358252 | 3.48 | 0.0869 |
| Oysters | 1 | 5.35046202 | 5.35046202 | 8.04 | 0.0150 |
| AmbTemp*Oysters | 1 | 8.54233143 | 8.54233143 | 12.84 | 0.0038 |
