# Supplementary material for: Seagrass and oyster interactions under a warming climate scenario: A mesocosm experiment
Source: PLoS One. 2025 Dec 11;20(12):e0337843. doi: 10.1371/journal.pone.0337843 (PMC12698006; doi:10.1371/journal.pone.0337843)
Supplement: S11a Table — Full model results from the GLM procedure. (DOCX) [file pone.0337843.s014.docx]

Supporting Information

S11a Table. (Log) chlorophyll *a* concentration at high tide across months. Full model results from the GLM procedure.

Dependent variable: (Log) chlorophyll *a* concentration at high tide across months.

| Source | DF | Sum of Squares | Mean Square | F Value | Pr > F |
| --- | --- | --- | --- | --- | --- |
| Model | 6 | 9.38271290 | 1.56378548 | 9.39 | <.0001 |
| Error | 25 | 4.16519297 | 0.16660772 |  |  |
| Corrected Total | 31 | 13.54790587 |  |  |  |

| R-Square | Coeff Var | Root MSE | lchl Mean |
| --- | --- | --- | --- |
| 0.692558 | 42.85721 | 0.408176 | 0.952409 |

| Source | DF | Type I SS | Mean Square | F Value | Pr > F |
| --- | --- | --- | --- | --- | --- |
| Amb_Temp | 1 | 0.75219461 | 0.75219461 | 4.51 | 0.0437 |
| Oysters | 1 | 0.04459015 | 0.04459015 | 0.27 | 0.6095 |
| month | 1 | 8.15411741 | 8.15411741 | 48.94 | <.0001 |
| month*Amb_Temp | 1 | 0.10838719 | 0.10838719 | 0.65 | 0.4275 |
| Amb_Temp*Oysters | 1 | 0.31765972 | 0.31765972 | 1.91 | 0.1796 |
| month*Oysters | 1 | 0.00576381 | 0.00576381 | 0.03 | 0.8539 |

| Source | DF | Type III SS | Mean Square | F Value | Pr > F |
| --- | --- | --- | --- | --- | --- |
| Amb_Temp | 1 | 0.75219461 | 0.75219461 | 4.51 | 0.0437 |
| Oysters | 1 | 0.04459015 | 0.04459015 | 0.27 | 0.6095 |
| month | 1 | 8.15411741 | 8.15411741 | 48.94 | <.0001 |
| month*Amb_Temp | 1 | 0.10838719 | 0.10838719 | 0.65 | 0.4275 |
| Amb_Temp*Oysters | 1 | 0.31765972 | 0.31765972 | 1.91 | 0.1796 |
| month*Oysters | 1 | 0.00576381 | 0.00576381 | 0.03 | 0.8539 |
