# Supplementary material for: Seagrass and oyster interactions under a warming climate scenario: A mesocosm experiment
Source: PLoS One. 2025 Dec 11;20(12):e0337843. doi: 10.1371/journal.pone.0337843 (PMC12698006; doi:10.1371/journal.pone.0337843)
Supplement: S11b Table — Full model results from the GLM procedure. (DOCX) [file pone.0337843.s015.docx]

Supporting Information

S11b Table. (Log) chlorophyll *a* concentration at low tide across months. Full model results from the GLM procedure.

Dependent variable: (Log) chlorophyll *a* concentration at low tide across months.

| Source | DF | Sum of Squares | Mean Square | F Value | Pr > F |
| --- | --- | --- | --- | --- | --- |
| Model | 6 | 1.90897696 | 0.31816283 | 1.23 | 0.3227 |
| Error | 25 | 6.44563706 | 0.25782548 |  |  |
| Corrected Total | 31 | 8.35461402 |  |  |  |

| R-Square | Coeff Var | Root MSE | lchl Mean |
| --- | --- | --- | --- |
| 0.228494 | 99.61314 | 0.507765 | 0.509737 |

| Source | DF | Type I SS | Mean Square | F Value | Pr > F |
| --- | --- | --- | --- | --- | --- |
| Amb_Temp | 1 | 0.34589644 | 0.34589644 | 1.34 | 0.2577 |
| Oysters | 1 | 0.48512775 | 0.48512775 | 1.88 | 0.1823 |
| month | 1 | 0.37118841 | 0.37118841 | 1.44 | 0.2414 |
| month*Amb_Temp | 1 | 0.35159750 | 0.35159750 | 1.36 | 0.2539 |
| Amb_Temp*Oysters | 1 | 0.21375312 | 0.21375312 | 0.83 | 0.3712 |
| month*Oysters | 1 | 0.14141374 | 0.14141374 | 0.55 | 0.4658 |

| Source | DF | Type III SS | Mean Square | F Value | Pr > F |
| --- | --- | --- | --- | --- | --- |
| Amb_Temp | 1 | 0.34589644 | 0.34589644 | 1.34 | 0.2577 |
| Oysters | 1 | 0.48512775 | 0.48512775 | 1.88 | 0.1823 |
| month | 1 | 0.37118841 | 0.37118841 | 1.44 | 0.2414 |
| month*Amb_Temp | 1 | 0.35159750 | 0.35159750 | 1.36 | 0.2539 |
| Amb_Temp*Oysters | 1 | 0.21375312 | 0.21375312 | 0.83 | 0.3712 |
| month*Oysters | 1 | 0.14141374 | 0.14141374 | 0.55 | 0.4658 |
