# Supplementary material for: Seagrass and oyster interactions under a warming climate scenario: A mesocosm experiment
Source: PLoS One. 2025 Dec 11;20(12):e0337843. doi: 10.1371/journal.pone.0337843 (PMC12698006; doi:10.1371/journal.pone.0337843)
Supplement: S12a Table — Full model results from the GLM procedure. (DOCX) [file pone.0337843.s016.docx]

**Supporting Information**

**S12a Table. Dissolved organic carbon (DOC) concentrations at high tide across months. Full model results from the GLM procedure.**

Dependent variable: DOC concentrations at high tide across sampling months.

| Source | DF | Sum of Squares | Mean Square | F Value | Pr > F |
| --- | --- | --- | --- | --- | --- |
| Model | 6 | 0.94462818 | 0.15743803 | 2.66 | 0.0392 |
| Error | 25 | 1.48181938 | 0.05927278 |  |  |
| Corrected Total | 31 | 2.42644756 |  |  |  |

| R-Square | Coeff Var | Root MSE | ldoc Mean |
| --- | --- | --- | --- |
| 0.389305 | 20.11046 | 0.243460 | 1.210614 |

| Source | DF | Type I SS | Mean Square | F Value | Pr > F |
| --- | --- | --- | --- | --- | --- |
| Amb_Temp | 1 | 0.46209978 | 0.46209978 | 7.80 | 0.0099 |
| Oysters | 1 | 0.08198365 | 0.08198365 | 1.38 | 0.2506 |
| month | 1 | 0.22253517 | 0.22253517 | 3.75 | 0.0640 |
| month*Amb_Temp | 1 | 0.02220895 | 0.02220895 | 0.37 | 0.5460 |
| Amb_Temp*Oysters | 1 | 0.00918603 | 0.00918603 | 0.15 | 0.6972 |
| month*Oysters | 1 | 0.14661460 | 0.14661460 | 2.47 | 0.1283 |

| Source | DF | Type III SS | Mean Square | F Value | Pr > F |
| --- | --- | --- | --- | --- | --- |
| Amb_Temp | 1 | 0.46209978 | 0.46209978 | 7.80 | 0.0099 |
| Oysters | 1 | 0.08198365 | 0.08198365 | 1.38 | 0.2506 |
| month | 1 | 0.22253517 | 0.22253517 | 3.75 | 0.0640 |
| month*Amb_Temp | 1 | 0.02220895 | 0.02220895 | 0.37 | 0.5460 |
| Amb_Temp*Oysters | 1 | 0.00918603 | 0.00918603 | 0.15 | 0.6972 |
| month*Oysters | 1 | 0.14661460 | 0.14661460 | 2.47 | 0.1283 |
