# Supplementary material for: Seagrass and oyster interactions under a warming climate scenario: A mesocosm experiment
Source: PLoS One. 2025 Dec 11;20(12):e0337843. doi: 10.1371/journal.pone.0337843 (PMC12698006; doi:10.1371/journal.pone.0337843)
Supplement: S12b Table — Full model results from the GLM procedure. (DOCX) [file pone.0337843.s017.docx]

**Supporting Information**

**S12b Table. Dissolved organic carbon (DOC) concentrations at low tide across months. Full model results from the GLM procedure.**

Dependent variable: DOC concentrations at low tide across sampling months.

| Source | DF | Sum of Squares | Mean Square | F Value | Pr > F |
| --- | --- | --- | --- | --- | --- |
| Model | 6 | 2.04977322 | 0.34162887 | 4.70 | 0.0025 |
| Error | 25 | 1.81771577 | 0.07270863 |  |  |
| Corrected Total | 31 | 3.86748900 |  |  |  |

| R-Square | Coeff Var | Root MSE | ldoc Mean |
| --- | --- | --- | --- |
| 0.530001 | 18.39067 | 0.269645 | 1.466208 |

| Source | DF | Type I SS | Mean Square | F Value | Pr > F |
| --- | --- | --- | --- | --- | --- |
| Amb_Temp | 1 | 0.04481342 | 0.04481342 | 0.62 | 0.4398 |
| Oysters | 1 | 0.01136304 | 0.01136304 | 0.16 | 0.6960 |
| month | 1 | 1.71103580 | 1.71103580 | 23.53 | <.0001 |
| month*Amb_Temp | 1 | 0.25927309 | 0.25927309 | 3.57 | 0.0706 |
| Amb_Temp*Oysters | 1 | 0.00982613 | 0.00982613 | 0.14 | 0.7163 |
| month*Oysters | 1 | 0.01346174 | 0.01346174 | 0.19 | 0.6707 |

| Source | DF | Type III SS | Mean Square | F Value | Pr > F |
| --- | --- | --- | --- | --- | --- |
| Amb_Temp | 1 | 0.04481342 | 0.04481342 | 0.62 | 0.4398 |
| Oysters | 1 | 0.01136304 | 0.01136304 | 0.16 | 0.6960 |
| month | 1 | 1.71103580 | 1.71103580 | 23.53 | <.0001 |
| month*Amb_Temp | 1 | 0.25927309 | 0.25927309 | 3.57 | 0.0706 |
| Amb_Temp*Oysters | 1 | 0.00982613 | 0.00982613 | 0.14 | 0.7163 |
| month*Oysters | 1 | 0.01346174 | 0.01346174 | 0.19 | 0.6707 |
