# Supplementary material for: Seagrass and oyster interactions under a warming climate scenario: A mesocosm experiment
Source: PLoS One. 2025 Dec 11;20(12):e0337843. doi: 10.1371/journal.pone.0337843 (PMC12698006; doi:10.1371/journal.pone.0337843)
Supplement: S13a Table — Full model results from the GLM procedure. [file pone.0337843.s018.docx]

**Supporting Information**

**S13a Table. Dissolved inorganic carbon (DIC) concentrations at high tide across months. Full model results from the GLM procedure.**

Dependent variable: DIC concentrations at high tide across sampling months.

| Source | DF | Sum of Squares | Mean Square | F Value | Pr > F |
| --- | --- | --- | --- | --- | --- |
| Model | 6 | 54.34035000 | 9.05672500 | 5.05 | 0.0016 |
| Error | 25 | 44.82090000 | 1.79283600 |  |  |
| Corrected Total | 31 | 99.16125000 |  |  |  |

| R-Square | Coeff Var | Root MSE | DIC Mean |
| --- | --- | --- | --- |
| 0.548000 | 5.765823 | 1.338968 | 23.22250 |

| Source | DF | Type I SS | Mean Square | F Value | Pr > F |
| --- | --- | --- | --- | --- | --- |
| Amb_Temp | 1 | 13.49401250 | 13.49401250 | 7.53 | 0.0111 |
| Oysters | 1 | 1.87695313 | 1.87695313 | 1.05 | 0.3160 |
| month | 1 | 15.55425312 | 15.55425312 | 8.68 | 0.0069 |
| month*Amb_Temp | 1 | 21.40215312 | 21.40215312 | 11.94 | 0.0020 |
| Amb_Temp*Oysters | 1 | 0.99052812 | 0.99052812 | 0.55 | 0.4642 |
| month*Oysters | 1 | 1.02245000 | 1.02245000 | 0.57 | 0.4572 |

| Source | DF | Type III SS | Mean Square | F Value | Pr > F |
| --- | --- | --- | --- | --- | --- |
| Amb_Temp | 1 | 13.49401250 | 13.49401250 | 7.53 | 0.0111 |
| Oysters | 1 | 1.87695313 | 1.87695313 | 1.05 | 0.3160 |
| month | 1 | 15.55425312 | 15.55425312 | 8.68 | 0.0069 |
| month*Amb_Temp | 1 | 21.40215312 | 21.40215312 | 11.94 | 0.0020 |
| Amb_Temp*Oysters | 1 | 0.99052812 | 0.99052812 | 0.55 | 0.4642 |
| month*Oysters | 1 | 1.02245000 | 1.02245000 | 0.57 | 0.4572 |
