# Supplementary material for: Seagrass and oyster interactions under a warming climate scenario: A mesocosm experiment
Source: PLoS One. 2025 Dec 11;20(12):e0337843. doi: 10.1371/journal.pone.0337843 (PMC12698006; doi:10.1371/journal.pone.0337843)
Supplement: S13b Table — Full model results from the GLM procedure. (DOCX) [file pone.0337843.s019.docx]

**Supporting Information**

**S13b Table. Dissolved inorganic carbon (DIC) concentrations at low tide across months. Full model results from the GLM procedure.**

Dependent variable: DIC concentrations at low tide across sampling months.

| Source | DF | Sum of Squares | Mean Square | F Value | Pr > F |
| --- | --- | --- | --- | --- | --- |
| Model | 6 | 38.88895938 | 6.48149323 | 4.45 | 0.0034 |
| Error | 25 | 36.43491250 | 1.45739650 |  |  |
| Corrected Total | 31 | 75.32387188 |  |  |  |

| R-Square | Coeff Var | Root MSE | DIC Mean |
| --- | --- | --- | --- |
| 0.516290 | 5.166057 | 1.207227 | 23.36844 |

| Source | DF | Type I SS | Mean Square | F Value | Pr > F |
| --- | --- | --- | --- | --- | --- |
| Amb_Temp | 1 | 9.69100313 | 9.69100313 | 6.65 | 0.0162 |
| Oysters | 1 | 0.03645000 | 0.03645000 | 0.03 | 0.8756 |
| month | 1 | 12.81445313 | 12.81445313 | 8.79 | 0.0066 |
| month*Amb_Temp | 1 | 14.24445313 | 14.24445313 | 9.77 | 0.0044 |
| Amb_Temp*Oysters | 1 | 1.06580000 | 1.06580000 | 0.73 | 0.4006 |
| month*Oysters | 1 | 1.03680000 | 1.03680000 | 0.71 | 0.4070 |

| Source | DF | Type III SS | Mean Square | F Value | Pr > F |
| --- | --- | --- | --- | --- | --- |
| Amb_Temp | 1 | 9.69100313 | 9.69100313 | 6.65 | 0.0162 |
| Oysters | 1 | 0.03645000 | 0.03645000 | 0.03 | 0.8756 |
| month | 1 | 12.81445313 | 12.81445313 | 8.79 | 0.0066 |
| month*Amb_Temp | 1 | 14.24445313 | 14.24445313 | 9.77 | 0.0044 |
| Amb_Temp*Oysters | 1 | 1.06580000 | 1.06580000 | 0.73 | 0.4006 |
| month*Oysters | 1 | 1.03680000 | 1.03680000 | 0.71 | 0.4070 |
