# Supplementary material for: Seagrass and oyster interactions under a warming climate scenario: A mesocosm experiment
Source: PLoS One. 2025 Dec 11;20(12):e0337843. doi: 10.1371/journal.pone.0337843 (PMC12698006; doi:10.1371/journal.pone.0337843)
Supplement: S14a Table — Full model results from the GLM procedure. (DOCX) [file pone.0337843.s020.docx]

Supporting Information

S14a Table. (Log) ammonia (NH_3_) concentration at high tide across months. Full model results from the GLM procedure.

Dependent variable: (Log) ammonia concentration at high tide across months.

| Source | DF | Sum of Squares | Mean Square | F Value | Pr > F |
| --- | --- | --- | --- | --- | --- |
| Model | 6 | 1.63352662 | 0.27225444 | 2.09 | 0.0903 |
| Error | 25 | 3.25332854 | 0.13013314 |  |  |
| Corrected Total | 31 | 4.88685516 |  |  |  |

| R-Square | Coeff Var | Root MSE | lnh3 Mean |
| --- | --- | --- | --- |
| 0.334269 | 20.18977 | 0.360740 | 1.786745 |

| Source | DF | Type I SS | Mean Square | F Value | Pr > F |
| --- | --- | --- | --- | --- | --- |
| Amb_Temp | 1 | 0.00000007 | 0.00000007 | 0.00 | 0.9994 |
| Oysters | 1 | 0.24575801 | 0.24575801 | 1.89 | 0.1816 |
| month | 1 | 0.55557107 | 0.55557107 | 4.27 | 0.0493 |
| month*Amb_Temp | 1 | 0.19091693 | 0.19091693 | 1.47 | 0.2371 |
| Amb_Temp*Oysters | 1 | 0.00032530 | 0.00032530 | 0.00 | 0.9605 |
| month*Oysters | 1 | 0.64095525 | 0.64095525 | 4.93 | 0.0358 |

| Source | DF | Type III SS | Mean Square | F Value | Pr > F |
| --- | --- | --- | --- | --- | --- |
| Amb_Temp | 1 | 0.00000007 | 0.00000007 | 0.00 | 0.9994 |
| Oysters | 1 | 0.24575801 | 0.24575801 | 1.89 | 0.1816 |
| month | 1 | 0.55557107 | 0.55557107 | 4.27 | 0.0493 |
| month*Amb_Temp | 1 | 0.19091693 | 0.19091693 | 1.47 | 0.2371 |
| Amb_Temp*Oysters | 1 | 0.00032530 | 0.00032530 | 0.00 | 0.9605 |
| month*Oysters | 1 | 0.64095525 | 0.64095525 | 4.93 | 0.0358 |
