# Supplementary material for: Seagrass and oyster interactions under a warming climate scenario: A mesocosm experiment
Source: PLoS One. 2025 Dec 11;20(12):e0337843. doi: 10.1371/journal.pone.0337843 (PMC12698006; doi:10.1371/journal.pone.0337843)
Supplement: S14b Table — Full model results from the GLM procedure. (DOCX) [file pone.0337843.s021.docx]

Supporting Information

S14b Table. (Log) ammonia (NH_3_) concentration at low tide across months. Full model results from the GLM procedure.

Dependent variable: (Log) ammonia concentration at low tide across months.

| Source | DF | Sum of Squares | Mean Square | F Value | Pr > F |
| --- | --- | --- | --- | --- | --- |
| Model | 6 | 2.07416594 | 0.34569432 | 0.69 | 0.6577 |
| Error | 25 | 12.47733384 | 0.49909335 |  |  |
| Corrected Total | 31 | 14.55149978 |  |  |  |

| R-Square | Coeff Var | Root MSE | lnh3 Mean |
| --- | --- | --- | --- |
| 0.142540 | 37.78279 | 0.706465 | 1.869807 |

| Source | DF | Type I SS | Mean Square | F Value | Pr > F |
| --- | --- | --- | --- | --- | --- |
| Amb_Temp | 1 | 0.27930049 | 0.27930049 | 0.56 | 0.4614 |
| Oysters | 1 | 0.13880787 | 0.13880787 | 0.28 | 0.6026 |
| month | 1 | 0.75650739 | 0.75650739 | 1.52 | 0.2297 |
| month*Amb_Temp | 1 | 0.10637729 | 0.10637729 | 0.21 | 0.6483 |
| Amb_Temp*Oysters | 1 | 0.79179857 | 0.79179857 | 1.59 | 0.2195 |
| month*Oysters | 1 | 0.00137433 | 0.00137433 | 0.00 | 0.9586 |

| Source | DF | Type III SS | Mean Square | F Value | Pr > F |
| --- | --- | --- | --- | --- | --- |
| Amb_Temp | 1 | 0.27930049 | 0.27930049 | 0.56 | 0.4614 |
| Oysters | 1 | 0.13880787 | 0.13880787 | 0.28 | 0.6026 |
| month | 1 | 0.75650739 | 0.75650739 | 1.52 | 0.2297 |
| month*Amb_Temp | 1 | 0.10637729 | 0.10637729 | 0.21 | 0.6483 |
| Amb_Temp*Oysters | 1 | 0.79179857 | 0.79179857 | 1.59 | 0.2195 |
| month*Oysters | 1 | 0.00137433 | 0.00137433 | 0.00 | 0.9586 |
