# Supplementary material for: Seagrass and oyster interactions under a warming climate scenario: A mesocosm experiment
Source: PLoS One. 2025 Dec 11;20(12):e0337843. doi: 10.1371/journal.pone.0337843 (PMC12698006; doi:10.1371/journal.pone.0337843)
Supplement: S15a Table — Full model results from the GLM procedure. (DOCX) [file pone.0337843.s022.docx]

**Supporting Information**

**S15a Table. (Log) nitrite (NO_2_) concentration at high tide across months. Full model results from the GLM procedure.**

Dependent variable: (Log) nitrite concentration at high tide across months.

| Source | DF | Sum of Squares | Mean Square | F Value | Pr > F |
| --- | --- | --- | --- | --- | --- |
| Model | 6 | 0.88287550 | 0.14714592 | 3.38 | 0.0139 |
| Error | 25 | 1.08702418 | 0.04348097 |  |  |
| Corrected Total | 31 | 1.96989968 |  |  |  |

| R-Square | Coeff Var | Root MSE | lni Mean |
| --- | --- | --- | --- |
| 0.448183 | -50.77359 | 0.208521 | -0.410688 |

| Source | DF | Type I SS | Mean Square | F Value | Pr > F |
| --- | --- | --- | --- | --- | --- |
| Amb_Temp | 1 | 0.00228382 | 0.00228382 | 0.05 | 0.8206 |
| Oysters | 1 | 0.10070447 | 0.10070447 | 2.32 | 0.1406 |
| month | 1 | 0.30379838 | 0.30379838 | 6.99 | 0.0140 |
| month*Amb_Temp | 1 | 0.02774074 | 0.02774074 | 0.64 | 0.4320 |
| Amb_Temp*Oysters | 1 | 0.05329161 | 0.05329161 | 1.23 | 0.2788 |
| month*Oysters | 1 | 0.39505647 | 0.39505647 | 9.09 | 0.0058 |

| Source | DF | Type III SS | Mean Square | F Value | Pr > F |
| --- | --- | --- | --- | --- | --- |
| Amb_Temp | 1 | 0.00228382 | 0.00228382 | 0.05 | 0.8206 |
| Oysters | 1 | 0.10070447 | 0.10070447 | 2.32 | 0.1406 |
| month | 1 | 0.30379838 | 0.30379838 | 6.99 | 0.0140 |
| month*Amb_Temp | 1 | 0.02774074 | 0.02774074 | 0.64 | 0.4320 |
| Amb_Temp*Oysters | 1 | 0.05329161 | 0.05329161 | 1.23 | 0.2788 |
| month*Oysters | 1 | 0.39505647 | 0.39505647 | 9.09 | 0.0058 |
