# Supplementary material for: Seagrass and oyster interactions under a warming climate scenario: A mesocosm experiment
Source: PLoS One. 2025 Dec 11;20(12):e0337843. doi: 10.1371/journal.pone.0337843 (PMC12698006; doi:10.1371/journal.pone.0337843)
Supplement: S15b Table — Full model results from the GLM procedure. (DOCX) [file pone.0337843.s023.docx]

Supporting Information

S15b Table. (Log) nitrite (NO_2_) concentration at low tide across months. Full model results from the GLM procedure.

Dependent variable: (Log) nitrite concentration at low tide across months.

| Source | DF | Sum of Squares | Mean Square | F Value | Pr > F |
| --- | --- | --- | --- | --- | --- |
| Model | 6 | 0.62272747 | 0.10378791 | 2.03 | 0.0998 |
| Error | 25 | 1.28098390 | 0.05123936 |  |  |
| Corrected Total | 31 | 1.90371137 |  |  |  |

| R-Square | Coeff Var | Root MSE | lni Mean |
| --- | --- | --- | --- |
| 0.327112 | -98.52457 | 0.226361 | -0.229751 |

| Source | DF | Type I SS | Mean Square | F Value | Pr > F |
| --- | --- | --- | --- | --- | --- |
| Amb_Temp | 1 | 0.16946197 | 0.16946197 | 3.31 | 0.0810 |
| Oysters | 1 | 0.00001350 | 0.00001350 | 0.00 | 0.9872 |
| month | 1 | 0.04067779 | 0.04067779 | 0.79 | 0.3814 |
| month*Amb_Temp | 1 | 0.33373919 | 0.33373919 | 6.51 | 0.0172 |
| Amb_Temp*Oysters | 1 | 0.00480238 | 0.00480238 | 0.09 | 0.7620 |
| month*Oysters | 1 | 0.07403263 | 0.07403263 | 1.44 | 0.2406 |

| Source | DF | Type III SS | Mean Square | F Value | Pr > F |
| --- | --- | --- | --- | --- | --- |
| Amb_Temp | 1 | 0.16946197 | 0.16946197 | 3.31 | 0.0810 |
| Oysters | 1 | 0.00001350 | 0.00001350 | 0.00 | 0.9872 |
| month | 1 | 0.04067779 | 0.04067779 | 0.79 | 0.3814 |
| month*Amb_Temp | 1 | 0.33373919 | 0.33373919 | 6.51 | 0.0172 |
| Amb_Temp*Oysters | 1 | 0.00480238 | 0.00480238 | 0.09 | 0.7620 |
| month*Oysters | 1 | 0.07403263 | 0.07403263 | 1.44 | 0.2406 |
