# Supplementary material for: Seagrass and oyster interactions under a warming climate scenario: A mesocosm experiment
Source: PLoS One. 2025 Dec 11;20(12):e0337843. doi: 10.1371/journal.pone.0337843 (PMC12698006; doi:10.1371/journal.pone.0337843)
Supplement: S16a Table — Full model results from the GLM procedure. (DOCX) [file pone.0337843.s024.docx]

Supporting Information

S16a Table. (Log) nitrate and nitrite (NO_x_) concentration at high tide across months. Full model results from the GLM procedure.

Dependent variable: (Log) NO_x_ concentration at high tide across months.

| Source | DF | Sum of Squares | Mean Square | F Value | Pr > F |
| --- | --- | --- | --- | --- | --- |
| Model | 6 | 7.05995951 | 1.17665992 | 2.69 | 0.0374 |
| Error | 25 | 10.93651507 | 0.43746060 |  |  |
| Corrected Total | 31 | 17.99647458 |  |  |  |

| R-Square | Coeff Var | Root MSE | lna Mean |
| --- | --- | --- | --- |
| 0.392297 | -264.4471 | 0.661408 | -0.250110 |

| Source | DF | Type I SS | Mean Square | F Value | Pr > F |
| --- | --- | --- | --- | --- | --- |
| Amb_Temp | 1 | 0.86191784 | 0.86191784 | 1.97 | 0.1727 |
| Oysters | 1 | 0.11146590 | 0.11146590 | 0.25 | 0.6181 |
| month | 1 | 5.93834616 | 5.93834616 | 13.57 | 0.0011 |
| month*Amb_Temp | 1 | 0.01458206 | 0.01458206 | 0.03 | 0.8566 |
| Amb_Temp*Oysters | 1 | 0.00000316 | 0.00000316 | 0.00 | 0.9979 |
| month*Oysters | 1 | 0.13364439 | 0.13364439 | 0.31 | 0.5854 |

| Source | DF | Type III SS | Mean Square | F Value | Pr > F |
| --- | --- | --- | --- | --- | --- |
| Amb_Temp | 1 | 0.86191784 | 0.86191784 | 1.97 | 0.1727 |
| Oysters | 1 | 0.11146590 | 0.11146590 | 0.25 | 0.6181 |
| month | 1 | 5.93834616 | 5.93834616 | 13.57 | 0.0011 |
| month*Amb_Temp | 1 | 0.01458206 | 0.01458206 | 0.03 | 0.8566 |
| Amb_Temp*Oysters | 1 | 0.00000316 | 0.00000316 | 0.00 | 0.9979 |
| month*Oysters | 1 | 0.13364439 | 0.13364439 | 0.31 | 0.5854 |
