# Supplementary material for: Seagrass and oyster interactions under a warming climate scenario: A mesocosm experiment
Source: PLoS One. 2025 Dec 11;20(12):e0337843. doi: 10.1371/journal.pone.0337843 (PMC12698006; doi:10.1371/journal.pone.0337843)
Supplement: S16b Table — Full model results from the GLM procedure. (DOCX) [file pone.0337843.s025.docx]

Supporting Information

S16b Table. (Log) nitrate and nitrite (NO_x_^-^) concentration at low tide across months. Full model results from the GLM procedure.

Dependent variable: (Log) NO_x_- concentration at low tide across months.

| Source | DF | Sum of Squares | Mean Square | F Value | Pr > F |
| --- | --- | --- | --- | --- | --- |
| Model | 6 | 16.41350854 | 2.73558476 | 2.22 | 0.0741 |
| Error | 25 | 30.73896594 | 1.22955864 |  |  |
| Corrected Total | 31 | 47.15247448 |  |  |  |

| R-Square | Coeff Var | Root MSE | lna Mean |
| --- | --- | --- | --- |
| 0.348094 | -199.2882 | 1.108855 | -0.556407 |

| Source | DF | Type I SS | Mean Square | F Value | Pr > F |
| --- | --- | --- | --- | --- | --- |
| Amb_Temp | 1 | 0.40836344 | 0.40836344 | 0.33 | 0.5696 |
| Oysters | 1 | 1.21711497 | 1.21711497 | 0.99 | 0.3293 |
| month | 1 | 14.57599504 | 14.57599504 | 11.85 | 0.0020 |
| month*Amb_Temp | 1 | 0.14214676 | 0.14214676 | 0.12 | 0.7367 |
| Amb_Temp*Oysters | 1 | 0.05476474 | 0.05476474 | 0.04 | 0.8346 |
| month*Oysters | 1 | 0.01512359 | 0.01512359 | 0.01 | 0.9126 |

| Source | DF | Type III SS | Mean Square | F Value | Pr > F |
| --- | --- | --- | --- | --- | --- |
| Amb_Temp | 1 | 0.40836344 | 0.40836344 | 0.33 | 0.5696 |
| Oysters | 1 | 1.21711497 | 1.21711497 | 0.99 | 0.3293 |
| month | 1 | 14.57599504 | 14.57599504 | 11.85 | 0.0020 |
| month*Amb_Temp | 1 | 0.14214676 | 0.14214676 | 0.12 | 0.7367 |
| Amb_Temp*Oysters | 1 | 0.05476474 | 0.05476474 | 0.04 | 0.8346 |
| month*Oysters | 1 | 0.01512359 | 0.01512359 | 0.01 | 0.9126 |
