# Supplementary material for: Seagrass and oyster interactions under a warming climate scenario: A mesocosm experiment
Source: PLoS One. 2025 Dec 11;20(12):e0337843. doi: 10.1371/journal.pone.0337843 (PMC12698006; doi:10.1371/journal.pone.0337843)
Supplement: S17a Table — Full model results from the GLM procedure. (DOCX) [file pone.0337843.s026.docx]

Supporting Information

S17a Table. (Log) orthophosphate (PO_4_^3^) concentration at high tide across months. Full model results from the GLM procedure.

Dependent variable: (Log) PO_4_^3^ concentration at high tide across months.

| Source | DF | Sum of Squares | Mean Square | F Value | Pr > F |
| --- | --- | --- | --- | --- | --- |
| Model | 6 | 2.08516293 | 0.34752716 | 5.40 | 0.0011 |
| Error | 25 | 1.60953597 | 0.06438144 |  |  |
| Corrected Total | 31 | 3.69469891 |  |  |  |

| R-Square | Coeff Var | Root MSE | lop Mean |
| --- | --- | --- | --- |
| 0.564366 | -197.9893 | 0.253735 | -0.128156 |

| Source | DF | Type I SS | Mean Square | F Value | Pr > F |
| --- | --- | --- | --- | --- | --- |
| Amb_Temp | 1 | 0.02216942 | 0.02216942 | 0.34 | 0.5626 |
| Oysters | 1 | 0.20493596 | 0.20493596 | 3.18 | 0.0865 |
| month | 1 | 1.06255481 | 1.06255481 | 16.50 | 0.0004 |
| month*Amb_Temp | 1 | 0.07336487 | 0.07336487 | 1.14 | 0.2960 |
| Amb_Temp*Oysters | 1 | 0.09459325 | 0.09459325 | 1.47 | 0.2368 |
| month*Oysters | 1 | 0.62754462 | 0.62754462 | 9.75 | 0.0045 |

| Source | DF | Type III SS | Mean Square | F Value | Pr > F |
| --- | --- | --- | --- | --- | --- |
| Amb_Temp | 1 | 0.02216942 | 0.02216942 | 0.34 | 0.5626 |
| Oysters | 1 | 0.20493596 | 0.20493596 | 3.18 | 0.0865 |
| month | 1 | 1.06255481 | 1.06255481 | 16.50 | 0.0004 |
| month*Amb_Temp | 1 | 0.07336487 | 0.07336487 | 1.14 | 0.2960 |
| Amb_Temp*Oysters | 1 | 0.09459325 | 0.09459325 | 1.47 | 0.2368 |
| month*Oysters | 1 | 0.62754462 | 0.62754462 | 9.75 | 0.0045 |
