# Supplementary material for: Seagrass and oyster interactions under a warming climate scenario: A mesocosm experiment
Source: PLoS One. 2025 Dec 11;20(12):e0337843. doi: 10.1371/journal.pone.0337843 (PMC12698006; doi:10.1371/journal.pone.0337843)
Supplement: S17b Table — Full model results from the GLM procedure. (DOCX) [file pone.0337843.s027.docx]

Supporting Information

S17b Table. (Log) orthophosphate (PO_4_^3^) concentration at low tide across months. Full model results from the GLM procedure.

Dependent variable: (Log) PO_4_^3^ concentration at low tide across months.

| Source | DF | Sum of Squares | Mean Square | F Value | Pr > F |
| --- | --- | --- | --- | --- | --- |
| Model | 6 | 0.86108850 | 0.14351475 | 5.09 | 0.0016 |
| Error | 25 | 0.70500431 | 0.02820017 |  |  |
| Corrected Total | 31 | 1.56609281 |  |  |  |

| R-Square | Coeff Var | Root MSE | lop Mean |
| --- | --- | --- | --- |
| 0.549832 | 162.8082 | 0.167929 | 0.103145 |

| Source | DF | Type I SS | Mean Square | F Value | Pr > F |
| --- | --- | --- | --- | --- | --- |
| Amb_Temp | 1 | 0.14253303 | 0.14253303 | 5.05 | 0.0336 |
| Oysters | 1 | 0.03356979 | 0.03356979 | 1.19 | 0.2857 |
| month | 1 | 0.13940458 | 0.13940458 | 4.94 | 0.0355 |
| month*Amb_Temp | 1 | 0.53800200 | 0.53800200 | 19.08 | 0.0002 |
| Amb_Temp*Oysters | 1 | 0.00276432 | 0.00276432 | 0.10 | 0.7568 |
| month*Oysters | 1 | 0.00481477 | 0.00481477 | 0.17 | 0.6830 |

| Source | DF | Type III SS | Mean Square | F Value | Pr > F |
| --- | --- | --- | --- | --- | --- |
| Amb_Temp | 1 | 0.14253303 | 0.14253303 | 5.05 | 0.0336 |
| Oysters | 1 | 0.03356979 | 0.03356979 | 1.19 | 0.2857 |
| month | 1 | 0.13940458 | 0.13940458 | 4.94 | 0.0355 |
| month*Amb_Temp | 1 | 0.53800200 | 0.53800200 | 19.08 | 0.0002 |
| Amb_Temp*Oysters | 1 | 0.00276432 | 0.00276432 | 0.10 | 0.7568 |
| month*Oysters | 1 | 0.00481477 | 0.00481477 | 0.17 | 0.6830 |
