# Supplementary material for: Seagrass and oyster interactions under a warming climate scenario: A mesocosm experiment
Source: PLoS One. 2025 Dec 11;20(12):e0337843. doi: 10.1371/journal.pone.0337843 (PMC12698006; doi:10.1371/journal.pone.0337843)
Supplement: S18a Table — Full model results from the GLM procedure. (DOCX) [file pone.0337843.s028.docx]

Supporting Information

S18a Table. Water temperature at high tide across months. Full model results from the GLM procedure.

Dependent variable: Water temperature at high tide across months.

| Source | DF | Sum of Squares | Mean Square | F Value | Pr > F |
| --- | --- | --- | --- | --- | --- |
| Model | 6 | 128.9418750 | 21.4903125 | 194.11 | <.0001 |
| Error | 25 | 2.7678125 | 0.1107125 |  |  |
| Corrected Total | 31 | 131.7096875 |  |  |  |

| R-Square | Coeff Var | Root MSE | WaterTemp  Mean |
| --- | --- | --- | --- |
| 0.978986 | 1.434395 | 0.332735 | 23.19688 |

| Source | DF | Type I SS | Mean Square | F Value | Pr > F |
| --- | --- | --- | --- | --- | --- |
| Amb_Temp | 1 | 20.0028125 | 20.0028125 | 180.67 | <.0001 |
| Oysters | 1 | 0.9453125 | 0.9453125 | 8.54 | 0.0073 |
| month | 1 | 107.6778125 | 107.6778125 | 972.59 | <.0001 |
| month*Amb_Temp | 1 | 0.2628125 | 0.2628125 | 2.37 | 0.1359 |
| Amb_Temp*Oysters | 1 | 0.0528125 | 0.0528125 | 0.48 | 0.4961 |
| month*Oysters | 1 | 0.0003125 | 0.0003125 | 0.00 | 0.9581 |

| Source | DF | Type III SS | Mean Square | F Value | Pr > F |
| --- | --- | --- | --- | --- | --- |
| Amb_Temp | 1 | 20.0028125 | 20.0028125 | 180.67 | <.0001 |
| Oysters | 1 | 0.9453125 | 0.9453125 | 8.54 | 0.0073 |
| month | 1 | 107.6778125 | 107.6778125 | 972.59 | <.0001 |
| month*Amb_Temp | 1 | 0.2628125 | 0.2628125 | 2.37 | 0.1359 |
| Amb_Temp*Oysters | 1 | 0.0528125 | 0.0528125 | 0.48 | 0.4961 |
| month*Oysters | 1 | 0.0003125 | 0.0003125 | 0.00 | 0.9581 |
