# Supplementary material for: Seagrass and oyster interactions under a warming climate scenario: A mesocosm experiment
Source: PLoS One. 2025 Dec 11;20(12):e0337843. doi: 10.1371/journal.pone.0337843 (PMC12698006; doi:10.1371/journal.pone.0337843)
Supplement: S18b Table — Full model results from the GLM procedure. (DOCX) [file pone.0337843.s029.docx]

Supporting Information

S18b Table. Water temperature at low tide across months. Full model results from the GLM procedure.

Dependent variable: Water temperature at low tide across months.

| Source | DF | Sum of Squares | Mean Square | F Value | Pr > F |
| --- | --- | --- | --- | --- | --- |
| Model | 9 | 299.0103125 | 33.2233681 | 26.01 | <.0001 |
| Error | 54 | 68.9840625 | 1.2774826 |  |  |
| Corrected Total | 63 | 367.9943750 |  |  |  |

| R-Square | Coeff Var | Root MSE | WaterTemp  Mean |
| --- | --- | --- | --- |
| 0.812540 | 4.884301 | 1.130258 | 23.14063 |

| Source | DF | Type I SS | Mean Square | F Value | Pr > F |
| --- | --- | --- | --- | --- | --- |
| Amb_Temp | 1 | 15.2100000 | 15.2100000 | 11.91 | 0.0011 |
| Oysters | 1 | 0.2500000 | 0.2500000 | 0.20 | 0.6600 |
| month | 2 | 269.4428125 | 134.7214063 | 105.46 | <.0001 |
| month*Amb_Temp | 2 | 13.9084375 | 6.9542187 | 5.44 | 0.0070 |
| Amb_Temp*Oysters | 1 | 0.1406250 | 0.1406250 | 0.11 | 0.7413 |
| month*Oysters | 2 | 0.0584375 | 0.0292188 | 0.02 | 0.9774 |

| Source | DF | Type III SS | Mean Square | F Value | Pr > F |
| --- | --- | --- | --- | --- | --- |
| Amb_Temp | 1 | 8.1450625 | 8.1450625 | 6.38 | 0.0145 |
| Oysters | 1 | 0.2480625 | 0.2480625 | 0.19 | 0.6612 |
| month | 2 | 269.4428125 | 134.7214063 | 105.46 | <.0001 |
| month*Amb_Temp | 2 | 13.9084375 | 6.9542187 | 5.44 | 0.0070 |
| Amb_Temp*Oysters | 1 | 0.1406250 | 0.1406250 | 0.11 | 0.7413 |
| month*Oysters | 2 | 0.0584375 | 0.0292188 | 0.02 | 0.9774 |
