# Supplementary material for: Seagrass and oyster interactions under a warming climate scenario: A mesocosm experiment
Source: PLoS One. 2025 Dec 11;20(12):e0337843. doi: 10.1371/journal.pone.0337843 (PMC12698006; doi:10.1371/journal.pone.0337843)
Supplement: S19a Table — Full model results from the GLM procedure. (DOCX) [file pone.0337843.s030.docx]

Supporting Information

S19a Table. Dissolved oxygen (DO) at high tide across months. Full model results from the GLM procedure.

Dependent variable: DO at high tide across months.

| Source | DF | Sum of Squares | Mean Square | F Value | Pr > F |
| --- | --- | --- | --- | --- | --- |
| Model | 6 | 57658.27250 | 9609.71208 | 11.88 | <.0001 |
| Error | 25 | 20230.13625 | 809.20545 |  |  |
| Corrected Total | 31 | 77888.40875 |  |  |  |

| R-Square | Coeff Var | Root MSE | DO_pct  Mean |
| --- | --- | --- | --- |
| 0.740268 | 19.47976 | 28.44654 | 146.0313 |

| Source | DF | Type I SS | Mean Square | F Value | Pr > F |
| --- | --- | --- | --- | --- | --- |
| Amb_Temp | 1 | 102.24500 | 102.24500 | 0.13 | 0.7252 |
| Oysters | 1 | 146.20500 | 146.20500 | 0.18 | 0.6744 |
| month | 1 | 56565.66125 | 56565.66125 | 69.90 | <.0001 |
| month*Amb_Temp | 1 | 73.20500 | 73.20500 | 0.09 | 0.7661 |
| Amb_Temp*Oysters | 1 | 22.11125 | 22.11125 | 0.03 | 0.8700 |
| month*Oysters | 1 | 748.84500 | 748.84500 | 0.93 | 0.3453 |

| Source | DF | Type III SS | Mean Square | F Value | Pr > F |
| --- | --- | --- | --- | --- | --- |
| Amb_Temp | 1 | 102.24500 | 102.24500 | 0.13 | 0.7252 |
| Oysters | 1 | 146.20500 | 146.20500 | 0.18 | 0.6744 |
| month | 1 | 56565.66125 | 56565.66125 | 69.90 | <.0001 |
| month*Amb_Temp | 1 | 73.20500 | 73.20500 | 0.09 | 0.7661 |
| Amb_Temp*Oysters | 1 | 22.11125 | 22.11125 | 0.03 | 0.8700 |
| month*Oysters | 1 | 748.84500 | 748.84500 | 0.93 | 0.3453 |
