# Supplementary material for: Seagrass and oyster interactions under a warming climate scenario: A mesocosm experiment
Source: PLoS One. 2025 Dec 11;20(12):e0337843. doi: 10.1371/journal.pone.0337843 (PMC12698006; doi:10.1371/journal.pone.0337843)
Supplement: S19b Table — Full model results from the GLM procedure. (DOCX) [file pone.0337843.s031.docx]

Supporting Information

S19b Table. Dissolved oxygen (DO) at low tide across months. Full model results from the GLM procedure.

Dependent variable: DO at low tide across months.

| Source | DF | Sum of Squares | Mean Square | F Value | Pr > F |
| --- | --- | --- | --- | --- | --- |
| Model | 9 | 61841.99094 | 6871.33233 | 11.52 | <.0001 |
| Error | 54 | 32221.54266 | 596.69523 |  |  |
| Corrected Total | 63 | 94063.53359 |  |  |  |

| R-Square | Coeff Var | Root MSE | DO_pct  Mean |
| --- | --- | --- | --- |
| 0.657449 | 19.25996 | 24.42735 | 126.8297 |

| Source | DF | Type I SS | Mean Square | F Value | Pr > F |
| --- | --- | --- | --- | --- | --- |
| Amb_Temp | 1 | 1313.15641 | 1313.15641 | 2.20 | 0.1438 |
| Oysters | 1 | 1717.06641 | 1717.06641 | 2.88 | 0.0956 |
| month | 2 | 51604.10016 | 25802.05008 | 43.24 | <.0001 |
| month*Amb_Temp | 2 | 5799.40266 | 2899.70133 | 4.86 | 0.0115 |
| Amb_Temp*Oysters | 1 | 510.19516 | 510.19516 | 0.86 | 0.3592 |
| month*Oysters | 2 | 898.07016 | 449.03508 | 0.75 | 0.4760 |

| Source | DF | Type III SS | Mean Square | F Value | Pr > F |
| --- | --- | --- | --- | --- | --- |
| Amb_Temp | 1 | 109.06506 | 109.06506 | 0.18 | 0.6707 |
| Oysters | 1 | 1362.47256 | 1362.47256 | 2.28 | 0.1366 |
| month | 2 | 51604.10016 | 25802.05008 | 43.24 | <.0001 |
| month*Amb_Temp | 2 | 5799.40266 | 2899.70133 | 4.86 | 0.0115 |
| Amb_Temp*Oysters | 1 | 510.19516 | 510.19516 | 0.86 | 0.3592 |
| month*Oysters | 2 | 898.07016 | 449.03508 | 0.75 | 0.4760 |
